# PKM2‐Driven Lactate Overproduction Triggers Endothelial‐To‐Mesenchymal Transition in Ischemic Flap via Mediating TWIST1 Lactylation

**DOI:** 10.1002/advs.202406184

**Published:** 2024-10-30

**Authors:** Yining Xu, Xianhui Ma, Weiyu Ni, Lin Zheng, Zhongnan Lin, Yingying Lai, Ningning Yang, Zhanqiu Dai, Teng Yao, Zeyang Chen, Lifeng Shen, Haitao Wang, Long Wang, Yizheng Wu, Weiyang Gao

**Affiliations:** ^1^ Department of Orthopaedics The Second Affiliated Hospital and Yuying Children's Hospital of Wenzhou Medical University Wenzhou 325027 China; ^2^ Zhejiang Provincial Key Laboratory of Orthopaedics Wenzhou 325027 China; ^3^ The Second Clinical Medical College of Wenzhou Medical University Wenzhou 325027 China; ^4^ Department of Orthopaedic Surgery Sir Run Run Shaw Hospital Zhejiang University School of Medicine Hangzhou 310003 China; ^5^ Key Laboratory of Musculoskeletal System Degeneration and Regeneration Translational Research of Zhejiang Province Hangzhou 310006 China

**Keywords:** endothelial‐to‐mesenchymal transition, lactate, lactylation, Pyruvate kinase M2, random‐pattern skin flap

## Abstract

The accumulation of lactate is a rising risk factor for patients after flap transplantation. Endothelial‐to‐mesenchymal transition (EndoMT) plays a critical role in skin fibrosis. Nevertheless, whether lactate overproduction directly contributes to flap necrosis and its mechanism remain unknown. The current study reveals that skin flap mice exhibit enhanced PKM2 and fibrotic response. Endothelial‐specific deletion of PKM2 attenuates flap necrosis and ameliorates flap fibrosis in mice. Administration of lactate or overexpressing PKM2 promotes dysfunction of endothelial cells and stimulates mesenchymal‐like phenotype following hypoxia. Mechanistically, glycolytic‐lactate induces a correlation between Twist1 and p300/CBP, leading to lactylation of Twist1 lysine 150 (K150la). The increase in K150la promotes Twist1 phosphorylation and nuclear translocation and further regulates the transcription of *TGFB1*, hence inducing fibrosis phenotype. Genetically deletion of endothelial‐specific PKM2 in mice diminishes lactate accumulation and Twist1 lactylation, then attenuates EndoMT‐associated fibrosis following flap ischemia. The serum lactate levels of flap transplantation patients are elevated and exhibit predictive value for prognosis. This findings suggested a novel role of PKM2‐derived lactate in mediating Twist1 lactylation and exacerbates flap fibrosis and ischemia. Inhibition of glycolytic‐lactate and Twist1 lactylation reduces flap necrosis and fibrotic response might become a potential therapeutic strategy for flap ischemia.

## Graphical abstract

Schematic illustration exhibiting mechanism of PKM2‐driven lactate overproduction‐induced EndoMT after flap ischemia: Abnormal expression of PKM2 promotes lactate overproduction of endothelial cell; Excessive lactate facilitates EndoMT and Twist1 lactylation of endothelial cell; Fibrotic response accelerates skin flap fibrosis and necrosis.

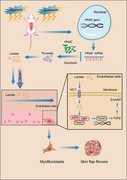

## 1 Introduction

Random‐pattern skin flaps are considered a feasible alternative in flap transplantation, which is a widely used strategy in traditional reconstructive surgery. However, these flaps are restrained by limited vascularity, as well as unbalanced homeostasis (when the flap length‐to‐width ratio surpasses 1.5‐2:1), and subject to distal necrosis, a common postoperative complication.^[^ ^1^ ^]^ Ischemia, triggered by degenerative microcirculation and inadequate blood supply, is a major pathological characteristic that causes such necroses. Once the progression of ischemia exceeds the tolerance threshold of the skin flap, ischemic necrosis commences, causing irreversible damage in the form of cell apoptosis, inflammation cascade, and fibrotic response.^[^ ^2^ ^]^ This leads to wound dehiscence and ultimate failure.

Endothelial‐to‐mesenchymal transition (EndoMT) is a cellular process wherein endothelial cells undergo a variety of molecular events and eventually assume mesenchymal‐like phenotypes similar to those of fibroblasts and myofibroblasts.^[^ ^3^, ^4^ ^]^ Evidently, EndoMT leads to a series of endothelial‐related diseases, such as pulmonary hypertension, renal fibrosis, myocardial infarction, and atherosclerosis.^[^ ^5^, ^6^, ^7^, ^8^, ^9^, ^10^ ^]^ Transforming growth factor–β (TGF‐β) plays a crucial role in EndoMT‐mediated fibrotic diseases.^[^ ^11^, ^12^, ^13^ ^]^ TGF‐β signaling may be stimulated by a series of factors, such as secreted cytokines and districted sheer stress.^[^ ^10^, ^14^ ^]^ Activated TGF‐β signaling induces the phosphorylation of Smad2/3, accompanied by the upregulated expression of EndoMT‐accelerating transcription factors, such as Twist1, which regulates EndoMT during vascular remodeling.^[^ ^15^, ^16^, ^17^, ^18^ ^]^

Abnormal glucose metabolism, especially aerobic glycolysis and the Warburg effect, are associated with metabolic disorders, which exert significant effects on various diseases.^[^ ^19^, ^20^, ^21^ ^]^ Even in blood vessels receiving sufficient oxygen supplies, dormant endothelial cells exhibit glycolysis‐addiction, because 85% of their required adenosine triphosphate (ATP) is generated glycolytically.^[^ ^22^, ^23^ ^]^ Glycolysis enables endothelial cells to avoid oxidative stress and provides an abundant metabolic supply to sustain vessel growth and angiogenesis.^[^ ^24^, ^25^ ^]^ This characteristic of quiescent endothelial cells that do not take advantage of enriched oxygen is analogous to the Warburg effect observed in tumor cells, which preferably utilize aerobic glycolysis to produce ATP and carbon production needed for cell proliferation and metastasis, even in an oxygen‐enriched atmosphere.^[^ ^26^ ^]^ Recent studies have shown that increased glycolysis accelerates EndoMT progression and cardiac endothelial cell dysfunction during cardiac fibrosis.^[^ ^27^ ^]^ Furthermore, it is widely acknowledged that glucose metabolism correlates firmly with fibrotic response, since glycolysis accounts for a major part of the energy required for collagen production and anabolism.^[^ ^28^ ^]^ However, the concerted mechanism underlying glycolysis in skin flaps has not been fully elucidated.

Pyruvate kinase M (PKM), which can be alternatively spliced into two isoforms, PKM1 and PKM2, acts as a key glycolytic enzyme that catalyzes the conversion of phosphoenolpyruvate to pyruvate and mediates the Warburg effect in cancers.^[^ ^29^, ^30^ ^]^ Additionally, PKM2 acts as a prominent isoform of pyruvate kinase that functions in endothelial cells and plays an essential role in endothelial cell proliferation.^[^ ^31^ ^]^ Moreover, PKM2 structurally exists as tetramer, dimer, or monomer. The tetrameric form maintains pyruvate kinase catalytic activity and facilitates mitochondria oxidative phosphorylation (OXPHOS), whereas the dimeric form exerts transcriptional function and protein kinase activity to enhance glycolysis.^[^ ^32^ ^]^ Besides, by interacting with multiple ligands or various post‐translational modifications (PTMs), such as acetylation, methylation and phosphorylation, the form of PKM2 varies from tetramer to dimer.^[^ ^33^ ^]^ PKM2‐mediated anaerobic glycolysis ultimately contributes to rapid lactate accumulation. Abundant endothelial cell‐derived lactate is released into peripheral circulation. Lactate, an important metabolite, promotes fibroblast differentiation, leading to pulmonary fibrosis.^[^ ^34^ ^]^ Recent studies have determined that glycolysis‐derived lactate directly modulates histones by adding lactyl groups to histone lysine (K) residues, a process which is termed “lactylation.^[^ ^35^ ^]^” However, the potential modulatory mechanism underlying the association between skin flap necrosis and endothelial cell‐derived lactate remains unclear. In this study, we found that enhanced endothelial cell‐derived lactate levels notably increased ischemic flap fibrosis and exacerbated flap necrosis by advancing EndoMT progression. Accordingly, lactate‐induced EndoMT and TGF‐β/Smad2 pathway activation was found to be remarkably attenuated in endothelial specific PKM2‐deletion mice. Mechanistically, endothelial cell‐derived lactate increased Twist1 nuclear translocation and lactylation following flap ischemia. Twist1 overexpression eliminated the improved phenotype mediated by *PKM2^fl/fl^*, *Tie2‐Cre* mice and further exacerbated EndoMT and skin flap necrosis. Our results indicate that serum lactate levels in transplant patients were strongly associated with the degree of ischemia. Furthermore, the expression levels of PKM2 and Twist1 were upregulated in patients with relatively poor prognoses. Therefore, we intend to discover the role of lactate in ischemic skin flaps and how it affects flap ischemia. Accordingly, we also aim to analyze whether serum lactate of flap transplantation patients could serve as an underlying diagnostic biomarker for ischemic flaps.

## 2 Results

### 2.1 Abnormal PKM2 Expression is Associated with Lactate Accumulation and EndoMT in Ischemic Flaps

**Figure 1:**
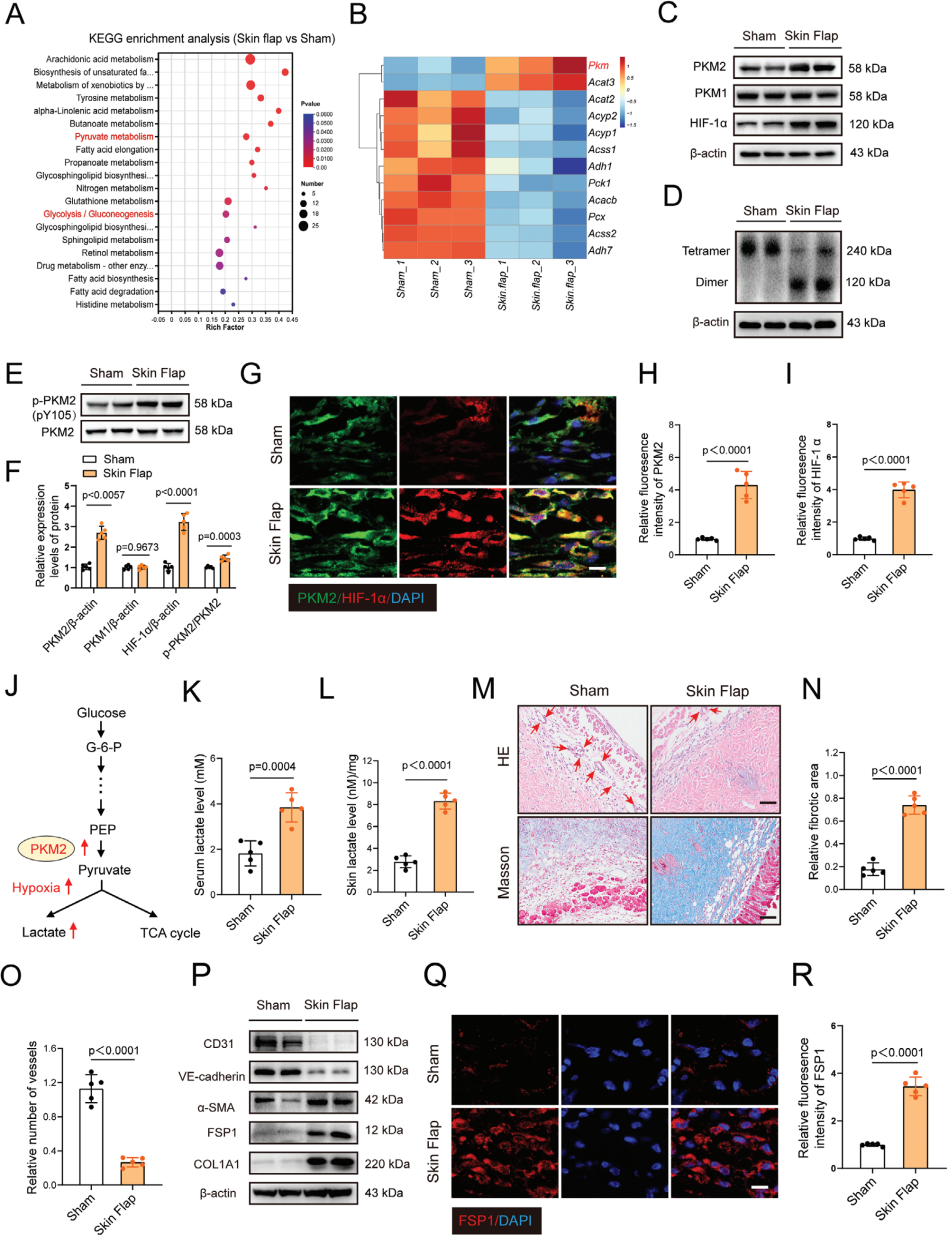
Enhanced expression of PKM2 is associated with lactate production and endothelial‐to‐mesenchymal transition in ischemic flaps. A) Metabolic pathway analysis exhibiting the top 20 pathways participated in differences between the skin from sham and skin flap mice. B) Heatmap showing differentially expressed genes involved in pyruvate metabolism in the skin from sham and skin flap mice. C) PKM1, PKM2 and HIF‐1α protein levels in the skin from sham and skin flap mice on postoperative day 7. D) Detection of dimeric and tetrameric PKM2 protein levels in the skin from sham and skin flap mice on postoperative day 7. E) Detection of p‐PKM2 (pY105) expression in the skin from sham and skin flap mice on postoperative day 7. F) Relative protein expression of PKM1, PKM2, HIF‐1α and p‐PKM2 in the skin from sham and skin flap mice on postoperative day 7. G) Immunofluorescence co‐staining of HIF‐1α and PKM2 of the dermal layer in the skin from sham and skin flap mice on postoperative day 7. Scale bar: 10 µm. H,I) Comparison of fluorescence intensity of HIF‐1α and PKM2 in the above two groups (n = 5). J) Schematic diagram of primary metabolic pathway for glucose flux through anaerobic glycolysis. K,L) Measurement of serum and skin lactate levels from sham and skin flap mice on postoperative day 7. (n = 5). M) Representative photomicrographs of H&E staining and Masson staining in the skin from sham and skin flap mice on postoperative day 7. Scale bar: 50 µm. N,O) Quantification of fibrotic area and number of vessels in the above two groups. (n = 5). P) Protein levels of endothelial marker CD31, VE‐cadherin, mesenchymal marker α‐SMA, Collagen1a1 and FSP1 in the skin from sham and skin flap mice on postoperative day 7. Q) Immunofluorescence staining of mesenchymal marker FSP1 in the skin from sham and skin flap mice on postoperative day 7. Scale bar: 10 µm. R) Comparison of fluorescence intensity of FSP1 in the above two groups (n = 5). Accurate *P*‐values are listed in the figures. Data is presented as mean±S.D. (F, H, I, K, L, N and R), unpaired two‐tailed *t* test.

To further explore the molecular mechanisms underlying ischemic flaps, RNA‐sequence analyses (RNA‐seq) of sham and random skin flaps were performed. The central part of the skin tissue from the sham and skin flap groups was extracted and subjected to RNA‐Seq. Principal component analysis (PCA) score plots (Figure S1A, Supporting Information) showed a prominent separation between the sham and skin flap groups. In addition, volcano plots and heatmaps (Figure S1B and C, Supporting Information) demonstrated significant transcriptional differences between these groups. To better understand the role of metabolic processes in ischemic flaps, we screened metabolism‐related pathways (**Figure** **1**A) using KEGG pathway analyses. The analyses revealed significant differences in pyruvate metabolism, as well as in glycolysis. *PKM* expression was notably upregulated among the various DEGS in pyruvate metabolism (Figure 1B). The pathology of ischemic flaps is often accompanied by ischemia and hypoxia.^[^ ^2^, ^36^ ^]^ Furthermore, hypoxia‐mediated metabolic transform is indispensable for attenuating mitochondrial respiration and stimulating glycolytic rate to maintain cellular energy.^[^ ^37^, ^38^ ^]^ Therefore, we further investigated the changes in *PKM* in ischemic flaps. Quantitative reverse transcription PCR (RT‐qPCR) enabled us to determine that the mRNA levels of hypoxia‐inducible factor 1α (*HIF‐1α*) and *PKM2* were significantly upregulated in the skin flap group, while the expression of *PKM1* remained unchanged (Figure S1D, Supporting Information). Furthermore, western blotting exhibited a corresponding increase in the protein levels of HIF‐1α and PKM2 in ischemic flaps (Figure 1C,F), while no obvious change was observed in the protein level of PKM1. Besides, we found that the tetrameric form of PKM2 was notably downregulated and replaced by dimeric PKM2 in skin flap mice (Figure 1D). Interestingly, phosphorylation of PKM2 tyrosine 105 (Y105) was significantly increased in skin flap mice, which accelerated PKM2 tetramer formation in ischemic flap (Figure 1E,F). The changes of PKM2 and HIF‐1α were also confirmed via immunofluorescence (IF) staining (Figure 1G–I). Lactate is the end product of glycolysis. Increased PKM2 catalyzes pyruvate synthesis and further promotes lactate accumulation under hypoxia (Figure 1J). We performed lactate measurement surveys to determine whether the ischemic flap elevates circulating lactate levels. Significantly, both serum and skin lactate levels increased after skin flap surgery, and this process was accompanied by flap necrosis (Figure 1K,L; Figure S2A and B, Supporting Information). Laser Doppler blood flow (LDBF) showed that the blood supply in the skin flap group was worse than that in the sham group (Figure S2C and D, Supporting Information). Collectively, these results indicated that abnormal glycolysis driven by PKM2 contributes to the development of ischemic flaps.

Previous investigations have confirmed that EndoMT tends to occur during pathological conditions involving hypoxia and fibroblast or myofibroblast accumulation.^[^ ^39^, ^40^ ^]^ We subsequently explored the specific role of EndoMT in ischemic flaps. Hematoxylin and eosin (H&E) as well as Masson staining indicated that ischemic skin flaps attenuated angiogenesis and promoted flap fibrosis in of mice (Figure 1M–O). In addition, we discovered that the ischemic flap markedly decreased the protein expression of the endothelial markers, VE‐cadherin and CD31 (Figure 1P). By contrast, the protein levels of mesenchymal markers, α‐smooth muscle actin (α‐SMA), fibroblast‐specific protein 1 (FSP1), and Collagen1a1 (COL1A1), in the skin flaps of mice were increased, compared with those of the sham group (Figure 1P). Accordingly, FSP1 staining in the ischemic flap was more marked, thereby revealing the progression of EndoMT in ischemic flaps (Figure 1Q,R). In addition, the degree of collagen damage was detected using 5‐FAM‐conjugated collagen‐hybridizing peptide (F‐CHP) staining. The results demonstrated that collagen damage in the skin flap group was significantly increased (Figure S2E and F, Supporting Information). These findings indicate a potential correlation between glycolysis and EndoMT in ischemic flaps.

### 2.2 PKM2 Regulates Glycolytic Lactate Accumulation and EndoMT in Endothelial Cells Following Hypoxia/Flap Ischemia

**Figure 2:**
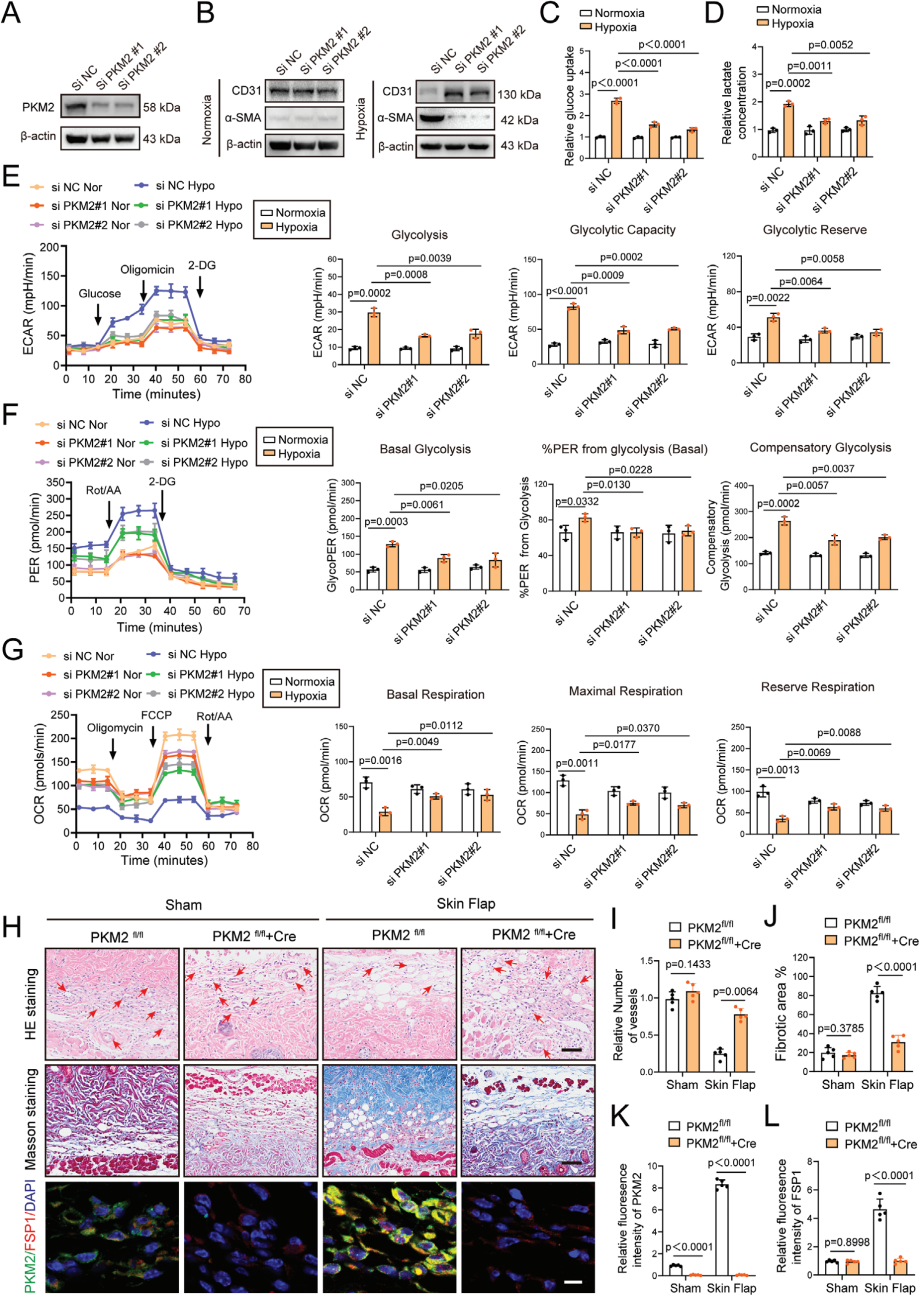
PKM2 regulates lactate production and EndoMT in endothelial cells following hypoxia. HUVECs were transfected with PKM2 siRNA after hypoxic or normoxic challenge. A) Knockdown efficiencies of PKM2 in HUVECs using western blotting. B) Western blotting detection of endothelial marker CD31 and mesenchymal marker α‐SMA under normoxia or hypoxia. C) PKM2 knockdown following hypoxia decreased glucose uptake in HUVECs (n = 3). D) PKM2 knockdown following hypoxia down‐regulated lactate production in HUVECs (n = 3). E–G) Glycolysis Stress, Glycolytic Rate and Mito Stress assays of HUVECs cultured and transfected as in panel. H) H&E staining, Masson staining and Immunofluorescence co‐staining of PKM2 and mesenchymal marker FSP1 from the above groups on postoperative day 7. I,J) Quantification of fibrotic area and number of vessels in the above four groups (n = 5). Scale bar: upper panel: 50 µm, middle panel: 50 µm, lower panel: 10 µm. K,L) Comparison of fluorescence intensity of FSP1 and PKM2 in the above four groups (n = 5). Accurate *P*‐values are listed in the figures. Data is presented as mean±S.D. C–G), Two‐way ANOVA; I–L), unpaired two‐tailed *t* test.

To evaluate the specific mechanism underlying the relationship between PKM2 and flap EndoMT that follows ischemia, PKM2 loss‐ and gain‐of‐function experiments were performed in vitro. Two PKM2 specific small interfering siRNAs were used to silence *PKM2* expression in human umbilical cord endothelial cells (HUVECs). Western blotting validated the knockdown efficiency of the PKM2 specific siRNAs (**Figure** **2**A). The results indicated that the expression of endothelial marker CD31, was notably decreased after hypoxic challenge, whereas that of the mesenchymal marker, α‐SMA, was upregulated (Figure 2B). PKM2 silencing significantly reversed these changes under hypoxic conditions, whereas no significant differences were detected under normoxic conditions (Figure 2B). Evidently, hypoxia leads to a strong increase in glycolysis. Consistently, hypoxia increased the acidification rate of the culture medium, as indicated by the color of the culture medium (Figure S3A, Supporting Information). However, PKM2 knockdown attenuated this change, indicating that acidic metabolite accumulation had been reduced (Figure S3A, Supporting Information). Hypoxic challenge significantly upregulated lactate production and glucose uptake of endothelial cells (Figure 2C,D). To investigate the specific mechanism of upregulated glucose uptake under hypoxia, we examined the expression of GLUT1 and GLUT3, which are widely distributed in the body and play the function of glucose transport. Accordingly, we confirmed that the protein expression of both GLUT1 and GLUT3 were elevated under hypoxia, which exerted vital impacts on glucose uptake (Figure S3B, Supporting Information). The above effects, which were induced by hypoxic conditions, were eliminated by PKM2 silencing (Figure 2C,D). In addition, seahorse assays were conducted to determine the effect of PKM2 under hypoxic conditions on glucose metabolism of endothelial cells. The glycolytic function was assessed by extracellular acidification rate (ECAR) (Figure 2E). Hypoxic challenge prominently elevated glycolysis level, maximum glycolytic capacity as well as glycolytic reserve in endothelial cells, while PKM2 knockdown eliminated these alterations. To diminish the impact of acidic metabolites generated by OXPHOS, glycolytic rate assays were performed. In accordance with the result of ECAR, hypoxia increased the glycolytic rate of endothelial cells, as evidenced by enhanced basal glycolysis, higher percentage of proton efflux rate form glycolysis and upregulated compensatory glycolysis (Figure 2F). Nevertheless, PKM2 silencing notably impaired the glycolytic rate. We further performed oxygen consumption rate (OCR) to detect the mitochondrial OXPHOS function in endothelial cells. As shown in Figure 2G, hypoxic treatment remarkably reduced mitochondrial respiration of endothelial cells. However, PKM2 knockdown significantly hindered the decrease of basal respiration, maximal respiration, and reserve respiratory capacity in endothelial cells under hypoxia (Figure 2G). Collectively, these metabolic data demonstrate that hypoxic challenge strengthens the glycolysis capacity of endothelial cells. While in the loss of PKM2, accompanied by the increase of PKM1/PKM2 ratio, the energetic metabolism of endothelial cells partially transforms from glycolysis to OXPHOS.^[^ ^41^ ^]^ During EndoMT biological process, endothelial cells eliminate cell‐cell junction characteristics and develop a capacity for migration and invasion thereby reaching adjacent tissues. Accordingly, migration assays were performed to examine the mesenchymal phenotype of endothelial cells. Hypoxia‐induced endothelial cell migration was reversed by PKM2 knockdown (Figure S3C, Supporting Information). We further explored whether hypoxia‐mediated EndoMT leads to endothelial cell dysfunction. Tube formation assay indicated that PKM2 downregulation rescued hypoxia‐mediated suppression of tube formation (Figure S3D, Supporting Information). Furthermore, collagen gel contraction assay demonstrated that endothelial cells exhibited an increased mesenchymal phenotype under hypoxic stimulation, which was attenuated by PKM2 knockdown as reflected by enhanced gel size and reduced contractility (Figure S3E, Supporting Information). Subsequently, we examined the direct regulatory effects of PKM2 on endothelial cells. A PKM2 overexpression plasmid was constructed and subsequently transfected into human umbilical vein endothelial cells (HUVECs) to upregulate PKM2 expression (Figure S4A and B, Supporting Information). Western blotting exhibited that under hypoxic conditions, enhanced PKM2 upregulated the expression of the mesenchymal marker, α‐SMA, while it reduced the expression of the endothelial cell marker, CD31 (Figure S4C and D, Supporting Information). Additionally, glucose uptake and lactate production of endothelial cells were notably upregulated by PKM2 overexpression under hypoxic conditions (Figure S4E–G, Supporting Information), suggesting that upregulated PKM2 accelerated endothelial cell‐derived lactate overproduction under hypoxia, then excessive lactate promoted fibrotic response to endothelial cells, thereby decreased the level of CD31 and enhanced the level of α‐SMA in endothelial cells. This finding was further confirmed by Seahorse analysis (Figure S4H–J, Supporting Information). Results of transwell, collagen gel contraction assay, and tube formation assays, indicated that PKM2 had accelerated endothelial cell dysfunction and EndoMT progression in endothelial cells affected by hypoxia (Figure S4K–M, Supporting Information). We investigated the specific role of PKM2 in vivo by establishing *PKM2^fl/fl^* mice, the genotypes of which were validated by PCR (Figure S4N, Supporting Information). To determine the function of PKM2 in the skin flap, *PKM2* conditional knockout mice with conditional deletion of the *PKM2* gene in endothelium, were generated employing Tie2‐Cre mice. Western blotting indicated that deletion of PKM2 in endothelial cells resulted in the upregulation of CD31 and downregulation of α‐SMA in ischemic skin flaps (Figure S3F, Supporting Information), which was further substantiated by H&E and Masson staining (Figure 2H–J). In addition, endothelial‐specific PKM2 knockout dramatically downregulated the serum lactate levels in skin flap mice (Figure S3G, Supporting Information). IF staining further confirmed that the reduction in the integrated intensity of PKM2 in the skin flap, caused by endothelial‐specific PKM2 deletion, was accompanied by a significant reduction in the expression of the mesenchymal marker, FSP1, indicating that the progression of EndoMT had been remarkably repressed (Figure 2H–L). Considered together, these data suggest that PKM2 facilitates EndoMT in the endothelial cells of skin flaps, following flap ischemia/hypoxia.

### 2.3 Excessive Lactate Accelerates EndoMT in Endothelial Cells after Hypoxia

**Figure 3:**
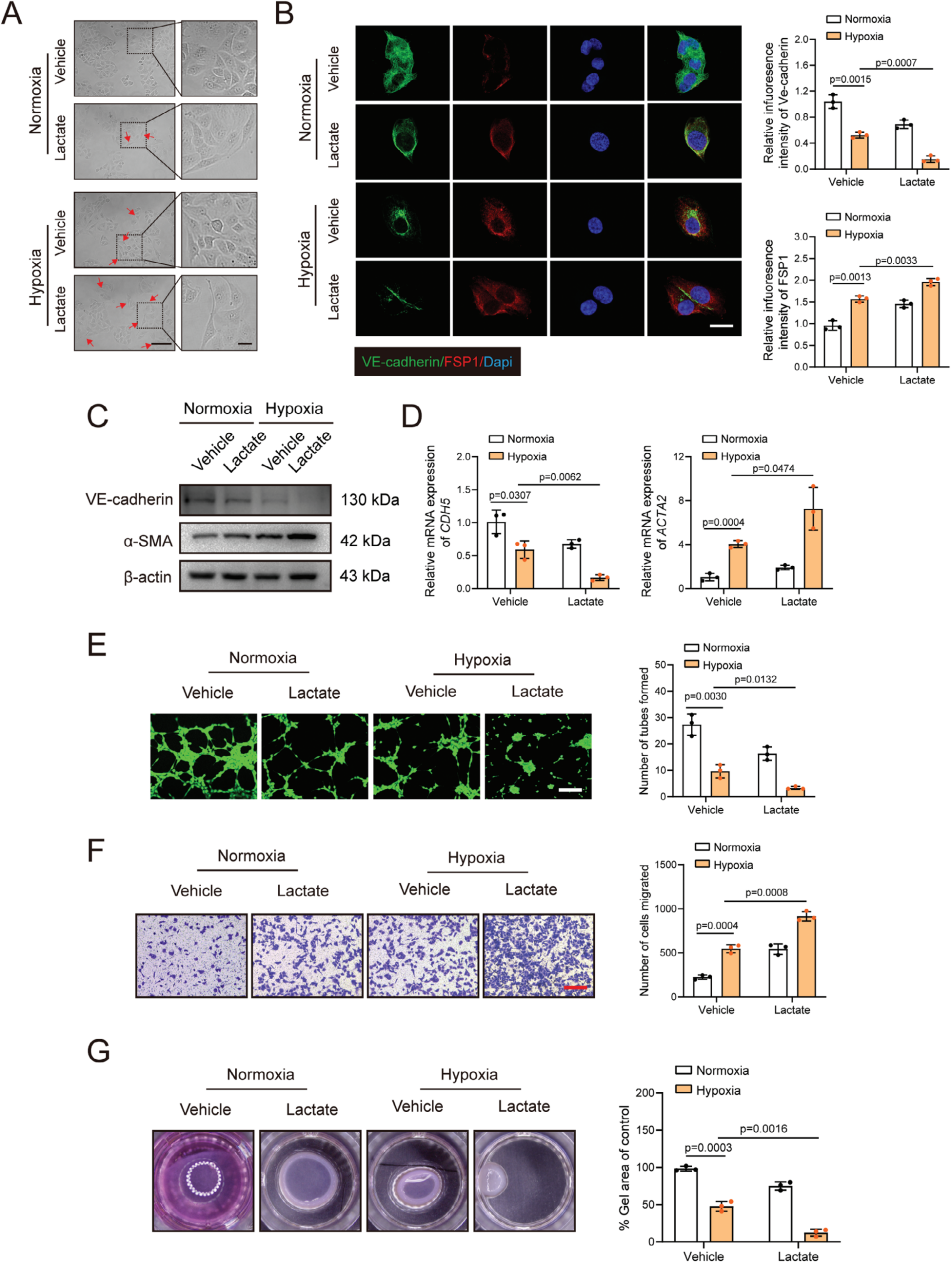
Supplemental lactate accelerates EndoMT progression in endothelial cells following hypoxia. HUVECs were cultured with 10 mM lactate after exposed to normoxia or hypoxia. A) Representative images of morphology of HUVECs. Scale bar: 50 µm, 25 µm. B) Immunofluorescence co‐staining of endothelial marker VE‐cadherin and mesenchymal marker FSP1 (n = 3). Scale bar: 25 µm. C) Western blotting measurement of mesenchymal marker α‐SMA and endothelial marker VE‐cadherin in endothelial cells. D) qRT‐PCR detection of mesenchymal marker α‐SMA and endothelial marker VE‐cadherin in endothelial cells. (n = 3). E) Angiogenesis of endothelial cells was determined by tube formation assay (n = 3). Scale bar: 200 µm. F) Migration of endothelial cells were detected using transwell assay (n = 3). Scale bar: 200 µm. G) Endothelial cell contractility was determined by collagen gel contraction assay (n = 3). Accurate *P*‐values are listed in the figures. Data is presented as mean±S.D. B and D–G), Two‐way ANOVA.

To identify the mechanisms underlying PKM2 induced promotion of EndoMT, lactate, the final product of anaerobic glycolysis, was added to the culture medium of endothelial cells under hypoxia. The proliferation and migration of endothelial cells were found to be strongly associated with EndoMT. Therefore, we next conducted transwell and EdU assays (**Figure** **3**F; Figure S5A, Supporting Information). Treatment with 10 mM lactate, rather than 5 mM lactate, markedly promoted endothelial cell proliferation following hypoxic challenge (Figure S5A, Supporting Information). Furthermore, the morphology of HUVECs changed from normal to a thread‐like spindle shape after culturing under hypoxic conditions for 48 h (Figure 3A). Stimulation with lactate led to more significant changes in morphology of endothelial cells than those in the hypoxic group (Figure 3A). Meanwhile, the elongated endothelial cells showed less VE‐cadherin expression and higher expression of the mesenchymal marker, FSP1 (Figure 3B). Accordingly, both mRNA and protein levels of α‐SMA were detected positively whereas those of VE‐cadherin were detected negatively in endothelial cells under hypoxic conditions (Figure 3C,D). Tube formation assays were performed to evaluate whether lactate‐induced EndoMT promoted endothelial cell dysfunction. The results showed that lactate suppressed endothelial cell‐based tube formation under hypoxic challenge (Figure 3E). Furthermore, both transwell and collagen gel contraction assays indicated that lactate‐administered endothelial cells showed an elevated mesenchymal phenotype following hypoxic challenge, as confirmed by the increased migration of cells and decreased collagen gel size (Figure 3F,G). Accordingly, we discovered that hypoxic challenge significantly downregulated the mRNA expression of *VEGFA* and *CDH5* and upregulated *ATCA2*, *S100A4*, and *COL1A1* levels in human dermal microvascular endothelial cells (HDMECs). Lactate administration further promoted these alterations in HDMECs (Figure S5B, Supporting Information). In summary, these results suggest that lactate promotes EndoMT after hypoxia.

### 2.4 Lactate Activates the TGF‐β/Smad2 Pathway Following Hypoxia

**Figure 4:**
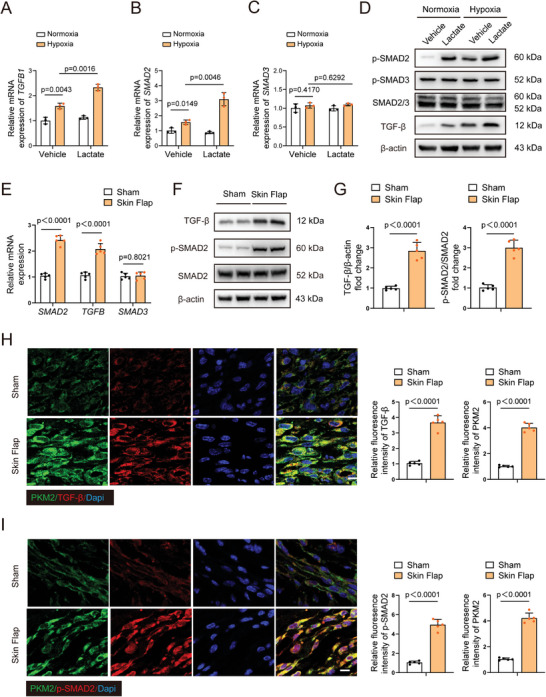
Lactate induces TGF‐β/Smad2 pathway following flap ischemia/hypoxia. A–D) HUVECs were stimulated with 10 mM lactate following exposed to normoxia or hypoxia. qRT‐PCR was employed to detect the mRNA levels of TGFB1, SMAD2 and SMAD3 (n = 3). Western blotting was conducted to measure the expression of TGF‐β, phospho(p)–SMAD2, phospho‐SMAD3. E–G) qRT‐PCR was employed to measure the mRNA expression of TGFB1, SMAD2 and SMAD3 in skin from sham and skin flap mice on postoperative day 7 (n = 5). Western blotting was employed to detect the protein levels of phospho‐SMAD2 and TGF‐β in the above groups (n = 5). H) Immunofluorescence co‐staining of PKM2 and TGF‐β in the above groups (n = 5). Scale bar, 10 µm. I) Immunofluorescence co‐staining of PKM2 and phospho‐SMAD2 in the above groups (n = 5). Scale bar, 10 µm. Accurate *P*‐values are listed in the figures. Data is presented as mean±S.D. (A‐C), Two‐way ANOVA; (E and G‐I), unpaired two‐tailed *t* test.

We investigated the mechanisms by which lactate regulates EndoMT progression after hypoxia. TGF‐β and Smad2/3 are known to play key roles in mediating EndoMT. In vitro qRT‐PCR results indicated that lactate had remarkably elevated *Smad2* and *Tgfβ1* mRNA level following hypoxia (**Figure** **4**A–C). However, the mRNA expression of *Smad3* remained unchanged. These results were also confirmed using HDMECs (Figure S5C, Supporting Information). Consistent with the alterations observed in mRNA expression, lactate stimulation increased the protein levels of TGF‐β and the phosphorylation of Smad2, but did not exert any effect on the phosphorylation of Smad3 following hypoxia (Figure 4D). We also detected that lactate accumulation had activated TGF‐β/Smad2 signaling in skin flap mice, compared to that in the control group (Figure 4E–I). Collectively, these results indicate that lactate induced TGF‐β/Smad2 activation may lead to EndoMT following hypoxia challenge.

### 2.5 Lactate Facilitates Twist1 Nuclear Translocation and Lactylation after Hypoxia

**Figure 5:**
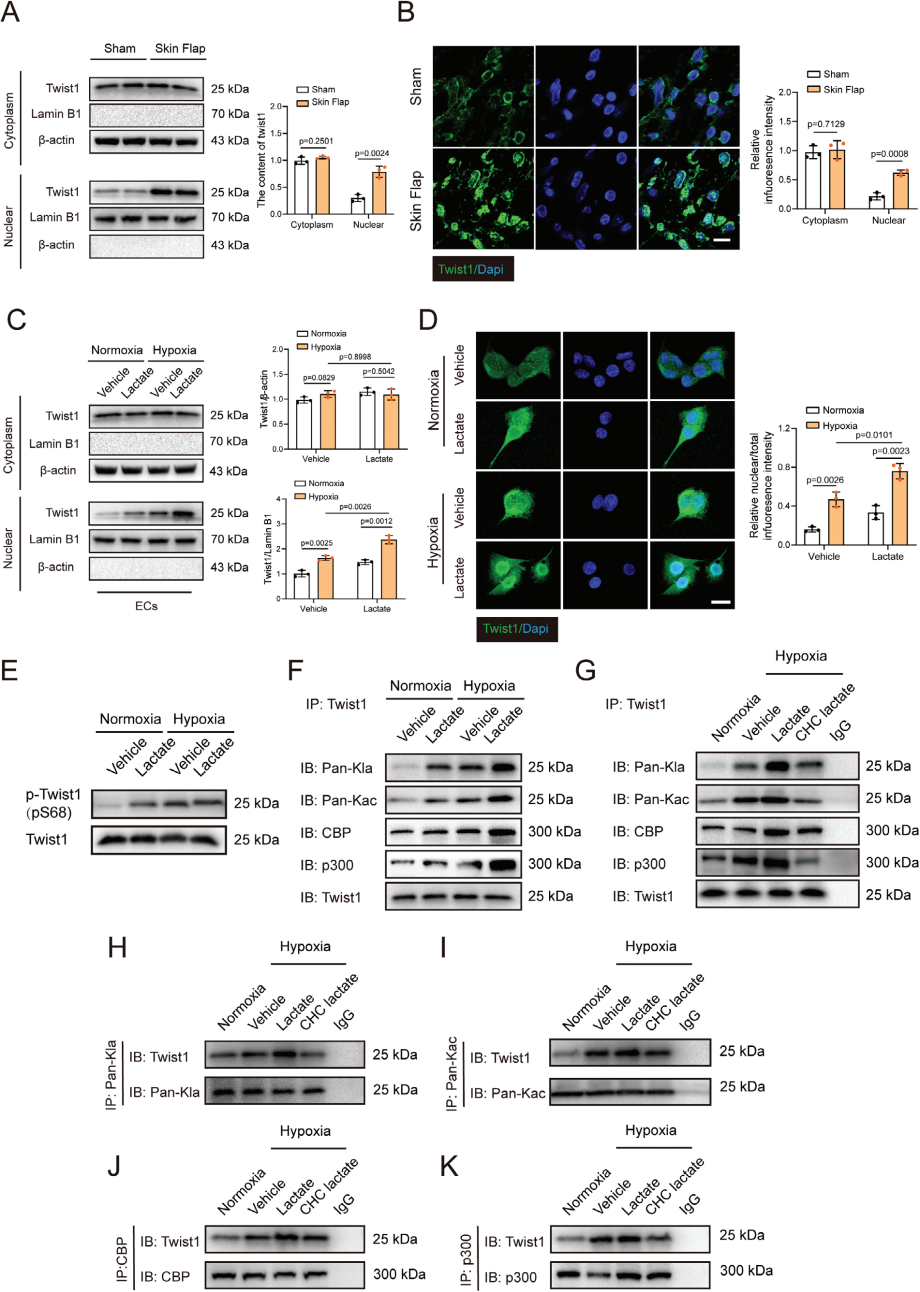
Lactate accelerates Twist1 nuclear translocation and lactylation after hypoxia. HUVECs were cultured with 10 mM lactate after exposed to normoxia or hypoxia. A,B) Western blotting and immunofluorescent staining were employed to detect the expression levels of cytoplasmic Twist1 and nuclear Twist1 in endothelial cells. Scale bar, 20 µm. C,D) Western blotting and immunofluorescent staining were employed to detect the expression levels of cytoplasmic Twist1 and nuclear Twist1 in skin from sham and skin flap mice on postoperative day 7 (n = 5). Scale bar, 10 µm. E) Western blotting was conducted to assess the expression of phospho(p)‐Twist1 (pS68) and Twist1. F) Lactylation and acetylation of Twist1, as well as the interaction between Twist1 and p300/CBP were detected using Immunoprecipitation (IP). G–K) MCT inhibitor CHC was utilized to inhibit intracellular lactate of endothelial cells before lactate treatment. Lactylation and acetylation of Twist1, and the interaction between Twist1 and p300/CBP were examined by immunoprecipitation. Accurate *P*‐values are listed in the figures. Data is presented as mean±S.D. A,B), unpaired two‐tailed *t* test; C,D), Two‐way ANOVA.

Twist1 is a transcription factor affiliated with the basic Helix‐Loop‐Helix (bHLH) family.^[^ ^42^ ^]^ Twist1 functions as an important transcriptional regulator of epithelial‐to‐mesenchymal transition (EMT).^[^ ^43^ ^]^ Hence, we explored the role played by Twist1 in lactate‐derived EndoMT via the TGF‐β/Smad2 pathway. In vivo western blotting and IF staining indicated that, the hypoxic conditions combined with lactate accumulation had induced Twist1 nuclear translocation in skin flap mice, compared with the sham group (**Figure** **5**A,B). Besides, hypoxia did not change the expression of the Twist1 cytoplasmic protein (Figure 5C). Nevertheless, the nuclear level of Twist1 was notably elevated following hypoxia compared to that in the normoxic group. Administration of lactate increased the nuclear translocation of Twist1. IF staining of nuclear Twist1 was heightened and became more intense following hypoxia, and this change was further increased by lactate stimulation (Figure 5D). Furthermore, we observed that hypoxia or lactate treatment upregulated the expression of phosphorylated Twist1 (pS68), suggesting that lactate could promote Twist1 nuclear translocation in an increasing Twist1 phosphorylation manner (Figure 5E). These experiments jointly demonstrated that nuclear translocation of Twist1 may play a vital role in lactate‐induced EndoMT.

Epigenetic modification serves as a key factor in the process of TGF‐β mediated EndoMT. Previous studies have verified that lactate directly modulates the transcription of homeostatic genes by inducing the lactylation of histone lysine residues in chromatin.^[^ ^35^ ^]^ Immunoprecipitation with anti‐Twist1 antibody was followed by immunoblotting using anti‐pan‐lactyl‐lysine (Kla) and anti‐pan‐acetyl‐lysine (Kac) antibodies, which showed that hypoxia promoted lactylation and acetylation of Twist1 in endothelial cells (Figure 5F). In addition, lactate treatment enhanced the lactylation and acetylation of Twist1. Previous studies have shown that Twist1 directly interacts with p300/CBP–associated factor (PCAF) causing p300 to promote its acetylation. Immunoprecipitation revealed an interaction between Twist1 and p300/CBP following hypoxia and subsequent lactate administration upregulated this interaction (Figure 5F). Monocarboxylate transporter (MCT) is a molecular transporter that bilaterally transports lactate across the plasma membrane. However, α‐cyano‐4‐hydroxycinnamate (CHC), an inhibitor of MCT, suppressed intracellular lactate, diminished intracellular lactate production following lactate administration (Figure S5D, Supporting Information), and impaired lactate‐derived interactions between p300/CBP as well as the lactylation and acetylation of Twist1 (Figure 5G). Immunoprecipitation with anti‐pan‐Kla antibody, anti‐pan‐Kac antibody, anti‐CBP antibody, and anti‐p300 antibody indicated that lactate administration notably induced the expression of Twist1, in comparison to that in the hypoxic challenge group, (Figure 5H–K). By contrast, CHC treatment dramatically decreased the interaction between Twist1 and p300/CBP as well as the acetylation and lactylation of Twist1. Subsequently, we examined the lactylation and acetylation of Twist1 in vivo. Similarly, the aggregation of lactate in ischemic flaps enhanced the lactylation and acetylation of Twist1, compared to those in the sham group (Figure S6A, Supporting Information). The lactylation and acetylation of Twist1 were downregulated in *PKM2^fl/fl^ Tie2‐Cre* mice, compared to that in mice of the *PKM2^fl/fl^* group (Figure S6B, Supporting Information). Additionally, deletion of endothelial‐specific PKM2 diminished the nuclear translocation of Twist1(Figure S6C, Supporting Information). Collectively, these data suggest that PKM2‐derived lactate induces the lactylation and acetylation of Twist1 under hypoxic conditions.

### 2.6 Lactate Promotes Activation of TGF‐β by Twist1 Nuclear Translocation

**Figure 6:**
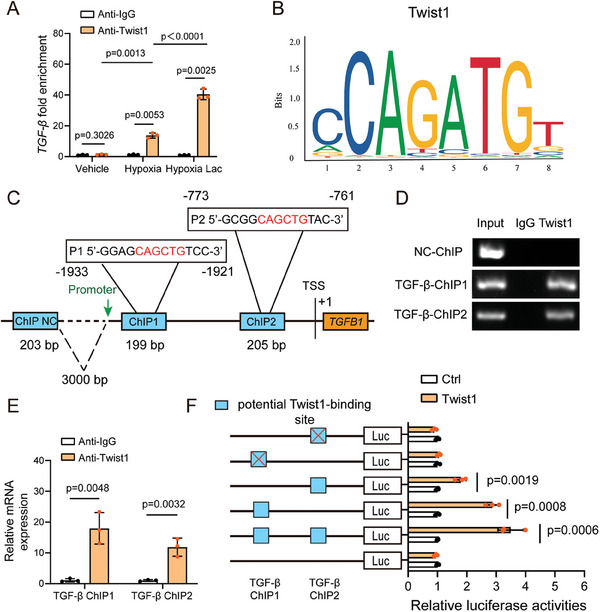
Lactate‐mediated Twist1 nuclear translocation in turn activates *TGFB1* expression. HUVECs were cultured with 10 mM lactate after exposed to normoxia or hypoxia. A) ChIP experiment was conducted with anti‐Twist1 antibody and subjected to qRT‐PCR utilizing primers specific for the promoter of TGF‐β in endothelial cells (n = 3). B) Transcription factor binding sites of Twist1 using JASPAR database. C,D) The direct binding of Twist1 to the *TGFB1* promoter was identified using ChIP assays in endothelial cells, including nonspecific control (ChIP NC) (a sequence located 3000 bp upstream from the transcription start site), *TGFB1*‐ChIP1 and *TGFB1*‐ChIP2. E) The direct binding capability of Twist1 to the *TGFB1* promoter was confirmed using qRT‐PCR in endothelial cells (n = 3). F) Dual‐luciferase reporter gene assay was employed to assess the regulation of Twist1 to *TGFB1* transcription in a ChIP 1 and ChIP 2 element‐dependent manner (n = 3). Accurate *P*‐values are listed in the figures. Data is presented as mean±S.D. A), Two‐way ANOVA; E,F), unpaired two‐tailed *t* test;.

To investigate whether upregulation of Twist1 nuclear localization contributes to lactate‐derived TGF‐β pathway activation, we assessed *TGFB1* recruitment levels by conducting chromatin immunoprecipitation (ChIP) experiments using anti‐Twist1 antibody. Twist1 and *TGFB1* levels in hypoxia‐challenged endothelial cells were significantly enriched (**Figure** **6**A). Furthermore, the combined *TGFB1* and the Twist1 protein expression in lactate‐stimulated hypoxia‐challenged endothelial cells was remarkably higher compared to that in cells exposed only to hypoxia (Figure 6A). Employing public bioinformatics database (JASPAR), we discovered that Twist1 possesses two putative binding sequences within the promoter region of the *TGFB1* gene, which were named as ChIP1 and ChIP2, and we selected a sequence 3000 bp upstream from the transcription start site (TSS) of *TGFB1* as a negative control (Figure 6B,C). Subsequently, we identified the direct binding of Twist1 to *TGFB1* using ChIP assay and qRT‐PCR in endothelial cells following hypoxia and lactate challenge (Figure 6D,E). ChIP 1, ChIP 2 and respective mutant sequences were established and inserted either separately or jointly into luciferase reporter gene plasmid to investigate the regulation of *TGFB1* expression by Twist1. The results of dual‐luciferase reporter gene experiment indicated that binding Twist1 to *TGFB1* activated the transcription of *TGFB1* in the manner of ChIP 1 and ChIP 2 element‐dependent (Figure 6F). Besides, mutations of both ChIP 1 and ChIP 2 elements abolished above activation. Together, these results indicate that lactate administration may induce an interaction between *TGFB1* and Twist1 protein, then activate the transcription of *TGFB1* by Twist1, thereby promoting EndoMT in skin flap via the TGF‐β/Smad2‐dependent pathway.

### 2.7 Twist1 Lysine 150 Lactylation Plays a Critical Role in EndoMT Progression of Endothelial Cells

**Figure 7:**
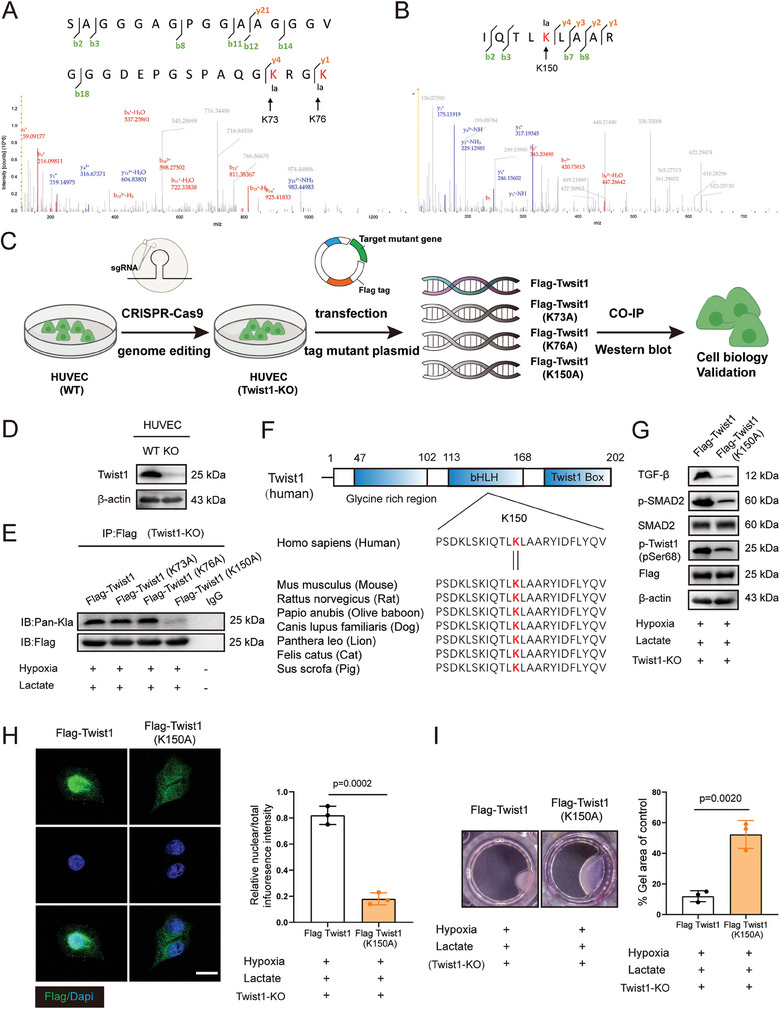
Twist1 K150 is the key site by which lactate drives Twist1 lactylation and exacerbates EndoMT. HUVECs were cultured with 10 mM lactate after exposed to hypoxia. A,B) Mass spectrometry spectrum of the lactylated Twist1 K73, K76 and K150 sites. C) Flowchart of in vitro experiments to validate lactate‐mediated lactylation of Twist1. D) Western blotting analysis of Twist1 knockout in endothelial cells. E) Western blotting analysis of Flag‐tag protein and Pan‐Kla expression levels after transfection of Flag‐Twist1, Flag‐Twist1 (K73A), Flag‐Twist1 (K75A) and Flag‐Twist1 (K150A) respectively. F) Schematic Representations of Twist1 protein domain and protein multiple sequence alignment analysis of Twist1 K150 sites. G) Western blotting analysis of TGF‐β, p‐SMAD2, SMAD2, p‐Twist1 (pSer68) and Flag‐tag. Twist1 KO endothelial cells was transfected with Flag‐Twist1 or Flag‐Twist1 (K150A) respectively. H) Under the same conditions as G), immunofluorescence staining of Flag‐tag (n = 3). Scale bar: 25 µm. I) Under the same conditions as G) Endothelial cell contractility was determined by collagen gel contraction assay (n = 3). Accurate *P*‐values are listed in the figures. Data is presented as mean±S.D. H,I), unpaired two‐tailed *t* test.

We subsequently explore the specific modification sites of Twist1 mediated by lactate. Endothelial cells were cultured under hypoxia following lactate administration. Lactylated proteins were pulled down using Twist1 antibody. Then immunoprecipitation‐mass spectrometry (IP‐MS) were employed. The assay results exhibited two Twist1 lactyl‐peptides, indicating that lactylation might occurred at the K73, K76 or K150 of Twist1 (**Figure** **7**A,B). To better elucidate the role of lactate‐driven lactylation of Twist1 in endothelial cells, first, we knock out Twist1 in HUVEC employing CRISPR/Cas9 gene editing technology to eliminate the effect of endogenous Twist1, then alanine was utilized to replace the lysine where lactylation might occur, and three single‐stie mutation plasmids and one wild‐type (WT) Twist1 plasmid tagged with Flag were constructed for further validation (Figure 7C). Western blotting results further identified the knockout efficiency of Twist1 (Figure 7D). Dramatically. according to the result of immunoprecipitation assay, we found that K150 mutation significantly attenuated the lactylation of Twist1 driven by lactate, while mutation of K73 and K76 exerted no obvious influence on Twist1 lactylation, revealing that K150 site was important in mediating Twist1 lactylation (Figure 7E). Furthermore, protein multiple sequence alignment comparison demonstrated that K150 is evolutionarily conserved among species (Figure 7F). Besides, as shown in schematic illustration, K150 is located in bHLH domain of Twist1, where enables Twist1 to regulate transcription and interact proteins (Figure 7F). Moreover, the activation of TGF‐β/Smad2 signaling was lower in endogenous Twist1 knockout endothelial cells transfected with Flag‐Twist1 K150A mutant plasmids than that of wild type following hypoxia and lactate treatment (Figure 7G). Besides, the level of phosphorylated Twist1 was obviously decreased in Twist1 K150A group (Figure 7G), and this discover was further confirmed by IF staining, as nuclear translocated Twsit1 was notably reduced in Twist1 K150A endothelial cells compared with WT group (Figure 7H). Furthermore, K150A remarkably abolished Twist1‐meidated endothelial cell contractility, as evidenced by collagen gel contraction assay (Figure 7I). Taken together, these data identified that lactylation at the K150 residue, but not at the K73 and K76 residues, contributes to the lactylation and nuclear translocation of Twist1 by lactate challenge.

### 2.8 Twist1 Inhibition Mitigates EndoMT Progression and TGF‐β/Smad2 Signaling Following Hypoxia

We further investigated whether administration of lactate accelerates EndoMT progression and activates TGF‐β/Smad2 signaling via Twist1 in vitro. Two Twist1 specific siRNAs were used to knockdown Twist1 expression, and their knockdown efficiency was further evaluated using qRT‐PCR (Figure S5E, Supporting Information). Si Twist1#2, which exhibited a higher knockdown efficiency, was selected for use in further studies. Intriguingly, the silencing of Twist1 significantly restored the decrease in VE‐cadherin and CD31 expression resulting from lactate treatment following hypoxia (Figure S7A and B, Supporting Information). On the contrary, silencing Twist1 attenuated the expression of lactate‐stimulated mesenchymal markers, FSP1 and α‐SMA (Figure S7A and B, Supporting Information). In addition, qRT‐PCR indicated that Twist1 inhibition upregulated the mRNA expression of *PECAM1*, *VEGFA* and *CDH5* (Figure S7C–E, Supporting Information), and downregulated the mRNA expression of *S100A4*, *ACTA2* and *COL1A1* following lactate administration (Figure S7F–H, Supporting Information). Furthermore, knockdown of Twist1 decreased *TGFB1* and *SMAD2* mRNA levels and accordingly attenuated the protein levels of TGF‐β and p‐SMAD2 in the presence of lactate stimulation (Figure S7I–L, Supporting Information).

### 2.9 Overexpression of Twist1 Promotes EndoMT and Impairs Endothelial‐Specific PKM2 Knockout‐Induced Ischemic Flap Survival

**Figure 8:**
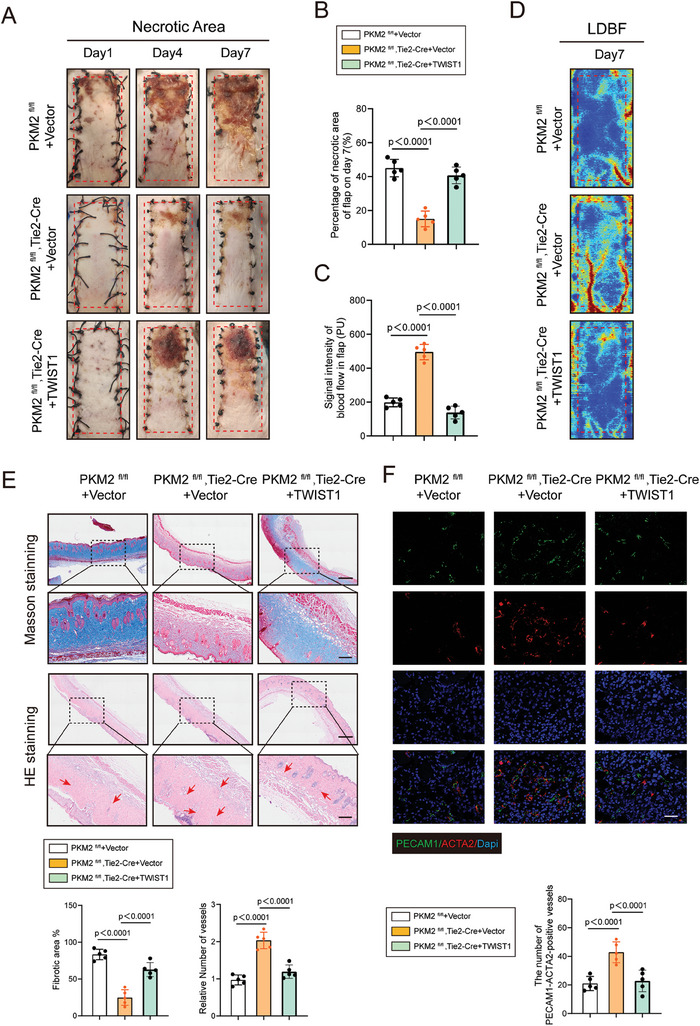
Overexpression of Twist1 accelerated EndoMT and necrosis in ischemic flap. *PKM2^fl/fl^*, and *PKM2^fl/fl^*, *Tie2‐Cre* mice were subjected to skin flap surgery and were sacrificed on postoperative day 7. A) Representative images of random‐pattern skin flaps on mice accompanied with Twist1 AAV or vector injection at different periods (1,4 and 7 days after operation). B) Analysis of the survival area of flaps on postoperative day 7 (n = 5). C,D) LDBF Photographing of skin flaps on postoperative day 7 (n = 5). E) Comparison of fibrotic area and number of vessels in the skin from skin flap mice accompanied with Twist1 AAV or vector injection on postoperative day 7 by Masson staining and H&E staining (n = 5). Scale bar: 500 µm, 100 µm. F) Analysis of PECAM1‐ACTA2‐positive blood vessels by immunofluorescence co‐staining of ACTA2 and PECAM1 in skin from the above groups (n = 5). Scale bar: 100 µm. Accurate *P*‐values are listed in the figures. Data is presented as mean±S.D. B,C and E,F), unpaired two‐tailed *t* test.

We further explored the role of Twist1 in PKM2‐mediated EndoMT in vitro. A PKM2 shRNA and a Twist1 overexpression plasmid were employed and their knockdown or overexpression efficiency in endothelial cells was measured using qRT‐PCR (Figure S8A and B, Supporting Information). Western blotting confirmed that upregulated Twist1 had reversed the increase in VE‐cadherin as well as the decrease in α‐SMA caused by silencing of PKM2 under hypoxia in endothelial cells (Figure S8C, Supporting Information). Moreover, the impaired mesenchyme‐like phenotype was detected in HUVECs overexpressing Twist1, as evidenced by Transwell and cell contraction assays (Figure S8D and E, Supporting Information). To confirm the role of Twist1 in ischemic flaps, Twist1 adeno‐associated virus (AAV) was used to overexpress Twist1. The efficiency of Twist1 overexpression was further evaluated via qRT‐PCR and IF staining for Twist1 (Figure S9A and B, Supporting Information). Twist1 AAV injection remarkably attenuated the survival of the ischemic flap (**Figure** **8**A,B). In addition, LDBF photographic images showed that Twist1 AAV impaired the improvement in blood supply mediated by PKM2 knockdown (Figure 8C,D). In addition, H&E and Masson staining revealed that Twist1 overexpression accelerated skin flap fibrosis and inhibited angiogenesis (Figure 8E). Furthermore, co‐staining of PECAM1 and ACTA2 confirmed that angiogenesis in the Twist1 AAV group had worsened (Figure 8F). Moreover, IF‐based co‐staining of PKM2 and FSP1 confirmed that Twsit1 overexpression dramatically diminished the suppression of EndoMT mediated by endothelial‐specific PKM2 deletion (Figure S9C and D, Supporting Information). In addition, Twist1 overexpression did not alter serum lactate levels in skin flap mice (Figure S9E, Supporting Information). Accordingly, IF staining and western blotting analysis revealed that Twist1 AAV had reactivated the TGF‐β/SMAD2 pathway following the loss of endothelial PKM2 (Figure S9F–H, Supporting Information). Collectively, these results suggested that Twist1 may act as a critical factor in PKM2‐mediated EndoMT by activating the TGF‐β/Smad2 pathway.

### 2.10 Serum Lactate is a Potential Ischemic Flap Prognosis Biomarker

**Figure 9:**
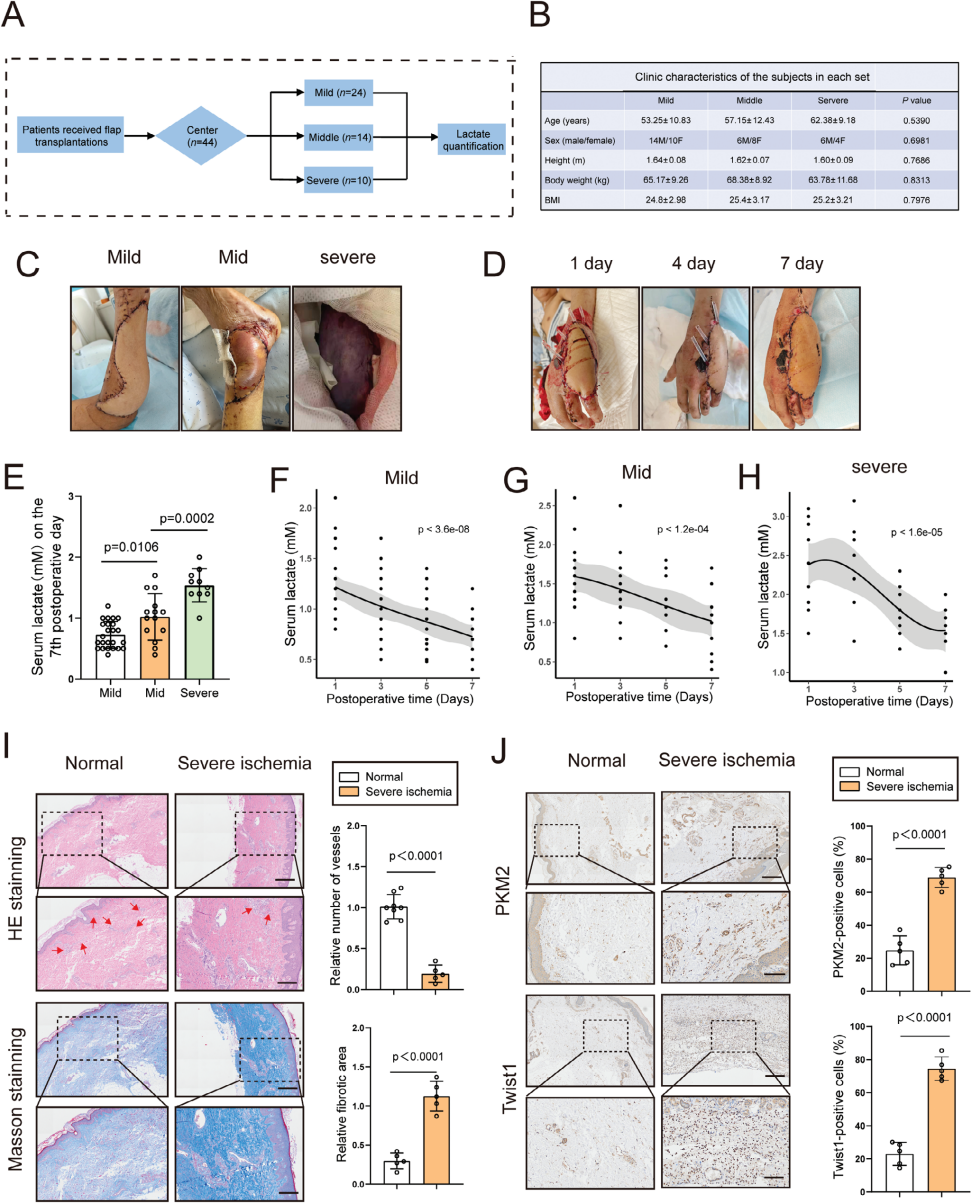
Serum lactate in patients is predictive for flap ischemia diagnosis. A) Schematic diagram indicating retrospective analysis for lactate in serum of patients with flap ischemia. B) Clinical analysis of patients including body weight, height, BMI, sex and age. C) Representative photographing of ischemic flaps with different degree. D) Representative photographing of ischemic flaps at different periods after flap transplantation (1, 4, 7 days). E) Serum lactate levels of patients with mild, middle and severe flap ischemia on postoperative day 7. F–H) Serum lactate levels were detected at 1, 3, 5, 7 days after flap transplantation. I) Comparison of fibrotic area and number of blood vessels in the skin from normal skin or severe ischemic skin flap utilizing H&E and Masson staining (n = 3). Scale bar: 500 µm, 200 µm. J) Immunohistochemistry of PKM2 and Twist1 in the skin from normal skin or severe ischemic skin flap (n = 3). Scale bar: 200 µm, 50 µm. Accurate *P*‐values are listed in the figures. Data is presented as mean±S.D. E and I,J), unpaired two‐tailed *t* test.

A retrospective study was conducted to investigate whether lactate in the serum of patients who underwent flap transplantation was upregulated (**Figure** **9**A). Under the inclusion criteria described in the Methods section, 48 patients who underwent skin transplantation were included in the study and divided into three groups (mild, mid, and severe ischemia); (Figure 9C). No significant variation was observed between age, sex, body weight, height or body mass index (BMI) (Figure 9B). Consistent with our hypothesis, serum lactate levels in patients with more severe flap ischemia were higher than those in patients who received better prognoses (Figure 9E). Furthermore, we discovered that serum lactate exhibited a gradually decreasing trend following flap transplantation, indicating that serum lactate levels were correlated with the postoperative recovery of patients (Figure 9D,F–H). In addition, we analyzed the histological differences between normal skin and skin showing severely ischemic flaps. Decreased blood vessels and enlarged fibrotic areas were detected in the ischemic flaps compared to those in normal skin tissue (Figure 9I). Notably, IHC indicated that increased expression of PKM2 and Twist1 in ischemic flaps was accompanied by enhanced fibrotic response (Figure 9J). Considered together, these data indicated that serum lactate levels may act as a biomarker of ischemic flaps.

## 3 Discussion

Skin flap transplantation, which involves a series of biological processes that take place during the postoperative period, is commonly recognized as an effective strategy for resolving large‐area cutaneous deficiencies in the field of clinical reconstructive surgery.^[^ ^1^, ^44^ ^]^ Nevertheless, ischemic damage and oxygen insufficiency are inevitable pathological conditions that closely affect the prognosis of skin flaps. Liu et al. discovered that HIF‐1α plays a pivotal role in hypoxia‐induced mesenchymal stem cell (MSC) mobilization and tissue repair.^[^ ^45^ ^]^ In the current study, we determined that PKM2‐derived lactate plays a novel role in the acceleration of ischemic flap fibrosis, by modulating EndoMT progression following flap ischemia. We confirmed that genetically deletion of endothelial‐specific PKM2 significantly ameliorates flap ischemia‐mediated EndoMT, flap fibrosis, and flap necrosis. By contrast, Twist1 overexpression enhanced EndoMT and promoted flap necrosis. Moreover, endothelial cell‐derived lactate promoted EndoMT in ischemic flap by activating TGF‐β/Smad2 signaling. By contrast, the knockdown of PKM2 and treatment with CHC, which function as a lactate transporter and MCT inhibitor, respectively, remarkably attenuated lactate‐derived EndoMT in endothelial cells following hypoxia. Notably, PKM2‐derived lactate predominantly promoted the lactylation of Twist1 lysine150 and facilitated nuclear translocation of Twist1 to directly regulate the transcription of *TGFB1* by binding to its promotor, thereby further enhancing the expression of TGF‐β. Silencing Twist1 mitigated lactate‐promoted EndoMT as well as TGF‐β/Smad2 signaling, following hypoxia. Similarly, Twist1 overexpression diminished flap ischemia and fibrosis mediated by the endothelial‐specific deletion of PKM2. Serum lactate levels in flap transplantation patients are considered as potential biomarkers of the prognosis for ischemic flaps. Collectively, these finding indicates that lactate, which had been recognized as a futile metabolite earlier, may indeed facilitate the pathophysiology of flap fibrosis, by accelerating EndoMT progression following flap ischemia. Therefore, targeting PKM2‐derived lactate accumulation may be regarded as a promising therapeutic strategy against flap ischemia. Thus, approaches that increase the elimination of excessive lactate in the circulation may improve the prognosis of patients who have undergone flap transplantation. Furthermore, the inhibition of lactate production in endothelial cells by targeting PKM2 may also enhance clinical treatment.

Lactate, which is generated during the final stage of anaerobic glycolysis, is recognized as a waste metabolite, compared to glucose.^[^ ^46^ ^]^ However, more recently, lactate has been attracting attention on account of its participation in a variety of biological processes. Lactate production increases when oxygen and ATP requirements are not met, such as during strenuous exercise and pathological ischemia.^[^ ^47^ ^]^ Emerging evidence indicates the potential of serum lactate as a molecular biomarker in various cardiac diseases, such as acute heart failure, heart transplantation, and myocardial infarction.^[^ ^8^, ^48^, ^49^, ^50^ ^]^ Moreover, enhanced scleral lactate may act as a molecular metabolite that promotes histone lactylation and facilitates fibroblast‐to‐myofibroblast transdifferentiation and myopia via H3K18la.^[^ ^51^ ^]^ In addition, lactate derived from endothelial cells regulates hippocampal neurogenesis in adults and osteogenesis in patients with osteoporosis.^[^ ^52^, ^53^ ^]^ However, studies that focus on abnormal glycolysis related lactate accumulation and its specific role in ischemic flaps are limited. In the current study, we confirmed that an increase in flap ischemia‐mediated lactate enhances flap necrosis and fibrosis in mice. By contrast, suppression of glycolysis‐lactate by genetically deleting endothelial‐specific PKM2 notably attenuated flap necrosis and fibrosis which follows flap ischemia. In vitro experiments have confirmed the role played by lactate in endothelial cell dysfunction and injury. These results indicate that glycolysis‐derived lactate mediates ischemic flap survival, by upregulating PKM2 expression.

PKM2 is an important isoform of pyruvate kinase that functions as the ultimate rate‐limiting enzyme in glycolysis.^[^ ^54^, ^55^ ^]^ Generally, PKM2 is highly expressed in tissues faced with increased anabolic demands, with particular reference to demands from proliferating cells, such as cancer cells.^[^ ^30^, ^56^, ^57^ ^]^ Excessively activated PKM2 induces lactate accumulation, disrupts glucose metabolism, and impairs homeostasis. Therefore, we hypothesized that PKM2 may participate in glycolysis‐derived lactate production in ischemic flaps. In the current study, we employed a genetically deletion of endothelial‐specific *PKM2* mouse model and discovered that PKM2 mediates angiogenesis, fibrosis, and necrosis following flap ischemia, by regulating glycolytic lactate production. Our in vitro data indicated that PKM2 overexpression increases lactate generation, attenuates angiogenesis, and strengthens contractility in endothelial cells, following hypoxia. These results suggest that PKM2 is highly expressed in ischemic flaps and contributes to endothelial cell dysfunction.

It is widely acknowledged that mesenchymal cells advance the progression of myofibroblasts and fibroblasts in numerous organs, including the skin. Dermal fibroblasts predominantly contribute to the development of skin fibrosis by converting into fibroblastic phenotypes. Conversely, activation of vascular endothelial cells attenuates dermal ischemia and injury. Previous studies have indicated that EndoMT may be involved in various cardiovascular and dermal diseases, including cardiac fibrosis, pulmonary hypertension, and systemic sclerosis.^[^ ^13^, ^58^, ^59^, ^60^ ^]^ Moreover, EndoMT promotes aortic remodeling and facilitates extravascular and intravascular fibrosis as well as microvascular rarefaction.^[^ ^11^, ^61^ ^]^ Enhanced HSP90α secretion by cells affected by EndoMT may further induce M2‐type macrophages to accelerate the proliferation and metastasis of pancreatic ductal adenocarcinoma.^[^ ^62^, ^63^ ^]^ Hence, we postulated that glycolysis‐derived lactate may lead to EndoMT following flap ischemia. To determine the mechanism by which EndoMT contributes to ischemic flap formation, we examined mesenchymal and endothelial markers following ischemic flap placement and found that increased glycolytic lactate levels promoted an elevated mesenchymal phenotype. Accordingly, repression of glycolysis‐derived‐lactate resulted in fewer endothelial cells transitioning to mesenchymal cells leading to attenuated flap fibrosis. These findings suggest that EndoMT serves as a central regulator of flap fibrosis following flap injury. Similar outcomes were observed in data gathered in vitro, confirming that administering exogenous lactate or increasing glycolysis‐derived lactate, by overexpressing PKM2, after hypoxia, upregulates mesenchymal markers and downregulates endothelial markers in endothelial cells. These findings indicate that endothelial lactate exacerbates flap fibrosis by accelerating EndoMT following flap ischemia.

Besides modulating fibrotic response in many fibrosis‐associated diseases, TGF‐β also functions as a cytokine which drives the EndoMT process.^[^ ^64^ ^]^ Smad2/3, which are downstream components of the TGF‐β signaling pathway, play a critical role in promoting cardiac fibrosis.^[^ ^65^ ^]^ The profibrogenic factor, TGF‐β, interacts with its receptor and further promotes the phosphorylation of Smad2/3 to activate the transcription of downstream genes. Furthermore, the deletion of Smad2/3 in cardiac fibroblasts effectively hindered fibrosis‐mediated gene expression and extracellular matrix remodeling. In the current study, we found that administering lactate or glycolysis‐derived lactate accelerates the activation of TGF‐β and Smad2, rather than the expression and phosphorylation of Smad3. Our results confirmed the participation of the TGF‐β/Smad2 pathway in endothelial lactate‐induced EndoMT following flap ischemia.

Twist1, which serves as a master transcription factor in EndoMT, participates in the pathological process via TGF‐β/Smad signaling.^[^ ^66^, ^67^ ^]^ Mechanistically, *Twist1* encodes a basic helix‐loop‐helix transcription factor that regulates target genes by directly interacting with their promoter regions via recognized enhancer‐box (E‐box) sequences.^[^ ^68^ ^]^ Analysis of the Human Protein Atlas indicated that Twist1 expression in dermal endothelial cells was higher than that in other skin cell types, such as fibroblasts. Additionally, nuclear translocation of Twist1 facilitates EMT, as evidenced by the finding that silencing genes upstream of Twist1 attenuates the EMT process.^[^ ^69^ ^]^ Hendee et al. revealed that endothelial Twist1 markedly contributes to age‐mediated decrease in blood vessel formation and regenerative lung development.^[^ ^66^ ^]^ The results of our current study confirmed that glycolytic lactate promotes the lactylation of Twist1 at the K150 residue, and facilitates the nuclear translocation of Twist1 in the manner of phosphorylation to regulate transcription of target gene. ChIP experiments indicated that nuclear Twist1 binds to the promoter of *TGFB1* to regulate transcription of TGF‐β. In addition, suppression of Twist1 inhibited EndoMT progression as well as EndoMT signaling following lactate administration and hypoxia, thereby indicating the prominent role played by Twist1 in the EndoMT process. Twist1 overexpression also promoted the mesenchymal phenotype in endothelial cells following hypoxia and PKM2 silencing, indicating that Twist1 may be a downstream target of PKM2 mediated by glycolytic lactate production. Furthermore, in vivo data indicated that Twist1 overexpression partially eliminated the amelioration of flap ischemia and fibrosis induced by Tie2‐specific PKM2 knockout. Recent findings indicate that lactate functions not only as a metabolic molecule but also as an epigenetic regulator and directly regulates the transcription of downstream targets.^[^ ^70^ ^]^ These findings suggest that intracellular lactate may play a significant role in epigenetic regulation. The histone acetylase, p300/CBP, recruits multiple molecules via a variety of protein‐binding domains and thereby facilitates the transcription of downstream targets.^[^ ^71^ ^]^ Recent studies have shown that Twist1 directly interacts with p300 and the p300/CBP–associated factor (PCAF), to influence histone acetyltransferase (HAT) activity.^[^ ^42^ ^]^ Accordingly, it was proven that PCAF acetylates Twist1 directly. We observed that administering lactate following hypoxia had enhanced the acetylation and lactylation of Twist1. Treatment with the MCT inhibitor, CHC, notably attenuated Twist1 lactylation and lactate acetylation. Accordingly, Tie2‐specific PKM2 knockout mice also exhibited downregulated lactylation and acetylation of Twist1 following flap ischemia. Mechanically, our finding indicates that glycolytic‐lactate‐induced Twist1 nuclear translocation and lactylation contributes to lactate‐mediated EndoMT by activating TGF‐β/Smad2 signaling after flap ischemia. In addition to our discovery, Zhang et al. found that hypoxia was a driving factor for histone lactylation.^[^ ^35^ ^]^ Therefore, histone lactylation may be considered a novel modification of the glycolytic lactate‐mediated EndoMT process following flap ischemia.

## 4 Conclusion

In summary, our study resulted in the elucidation of an undetermined role played by glycolysis‐derived lactate in accelerating EndoMT following flap ischemia/hypoxia. Glycolysis‐derived lactate stimulates the nuclear translocation and lactylation of Twist1, which in turn promotes EndoMT progression by activating the TGF‐β/Smad2 pathway. These findings offer a previously unrealized perspective on glycolysis‐derived lactate which indicates that it plays a role in the dysfunction of endothelial cells and shows potential as an important metabolic biomarker as well as a contributor to flap ischemia‐induced fibrosis and necrosis.

## 5 Experimental Section

### Human Tissue Sample Collection

All the procedures in study were approved by the Ethics Committee of the Second Affiliated Hospital and Yuying Children's Hospital of Wenzhou Medical University (WYDW2020‐0167) and conducted according to the Declaration of Helsinki. Written informed consent was obtained from patients before the beginning of this study. Human flap tissue and serum samples were acquired from patients who underwent flap transplantation surgeries at the Second Affiliated Hospital and Yuying Children's Hospital of Wenzhou Medical University between 2020 and 2023. Normal human skin tissue samples were obtained from patients who had undergone traumatic injury. Patients with cardiovascular or autoimmune diseases, such as hypertension, diabetes, hyperlipidemia, systemic sclerosis, and various other diseases that could affect skin flaps, were excluded from this study. Patients who underwent flap transplantation surgery were divided into three groups according to the degree of ischemia, necrotic area of the flaps, and other postoperative complications. Detailed criteria utilized for this classification were as follows: the mild ischemia group – good blood circulation of the flaps and the absence of complications; The mid ischemia group – flaps showed moderate arterial supply and venous drainage, along with areas of bruises and necrosis of < 10%, but did not require secondary surgical intervention; and the severe ischemia group – flaps showed bruised and necrotic areas exceeding the moderate range (> 10%), and required surgical exploration and intervention.

### Experimental Animal Studies

All animal experimental and operating procedures conducted in this study were approved by the Institutional Animal Care and Use Committee, basing on the approval of the Animal Care and Use Committee of Wenzhou Medical University (WYDY2023‐0587). The PKM2 conditionally knocked out mice, *PKM2^fl/fl^* (Strain NO. T053274) and Tie2‐Cre transgenic mice (Strain NO. T003764) were purchased from GemPharmatech (Nanjing, China). Briefly, mice with PKM2 conditionally knocked out in endothelial cells were generated by crossing *PKM2^fl/fl^* mice with Tie2‐Cre mice. And the genotypes were confirmed by PCR on genomic DNA. Protocols and primer sequences are accessible upon request.

For random‐pattern skin flaps model, a regular pedicled flap (size: 4.5 cm× 1.5 cm) was generated dorsally on mice, accompanied with the section of both sacral arteries. Sham mice were sham‐operated via preserving blood vessels. Experimental mice were photographed and handled and euthanized on day 7 following surgery.

The adeno‐associated virus (AAV) AAV‐Twist1 and AAV‐Vector employed in this study were purchased from HanbioTech (Shanghai, China). In brief, one month prior to skin flap surgery, in total of 16 µl AAV (≈5× 10^9^ plaque‐forming units (PFUs)) was subcutaneously injected utilizing a microsyringe. For further detection, the mice were sacrificed 7 days after surgery, and the flap samples were collected.

### RNA Sequencing and Bioinformatics Analysis

RNA‐seq analyses were performed to produce a profiling database of skin flaps. Briefly, total RNA was extracted from the skin tissue of three sham mice and three skin flap mice on day 7 following the operation utilizing TRIzol (Invitrogen). RNA sequencing was further performed by Majorbio (Shanghai, China). Differential Expression Genes (DEGs) were visualized and exhibited in volcano plots. KEGG bioinformatics analysis was performed using KOBAS database (http://kobas.cbi.pku.edu.cn/home.do). The BH (FDR) method was used for multiple tests to control the false positive rate, and the corrected P‐value was 0.05 as the threshold. KEGG pathways meeting this condition were defined as KEGG pathways that were significantly enriched in different‐expressed genes. The bubble colors from blue to red indicate that the p‐value of the relevant pathway decreases in turn. Besides, the larger the bubble is, the more related DEGs accumulate on the pathway.

### LDBF Photographing

To better present the visualization of vascular network of the skin flap, LDBF was employed in this study. Blood supply was measured 7 days after skin flap or sham operation using laser Doppler instruments (Axminster, UK). The assessments of blood supply were calculated using moorLDI system. Each mouse was subject to three repeat measurements, from which the mean value was utilized for further analysis.

### Cell Culture

In present study, the human umbilical vein endothelial cell lines (HUVEC) were acquired directly from the American‐Type Culture Collection and were subsequently cultured in Dulbecco's Modified Eagle Medium (DMEM) supplemented with 10% fetal bovine serum (FBS, GIBCO, Thermo Fischer Scientific), endothelial cell growth supplement (ECGS, 25 µg mL^−1^) and 1% antibiotics (penicillin‐streptomycin, Beyotime). Human dermal microvascular endothelial cells (HDMEC) were obtained from Haixing Biosciences (Suzhou, China) and were cultured in endothelial cell complete medium. Endothelial cells were treated with lactate solution (IC_50_: 25 mM, MCE, China) and were placed in the incubator with 5% CO_2_ at 37 °C. For hypoxic challenge, endothelial cells were kept in a hypoxia chamber containing 1%O_2_ and 5% CO_2_ at 37 °C for 48 h.

For small interfering RNA transfection, specific siRNAs (RiboBio, Guangzhou, China) were employed for the transfection of endothelial cells. Respectively, RNA and protein of endothelial cells were extracted 24 and 48 h following transfection. The sequences of employed siRNAs were listed in Table S1 (Supporting Information).

For lentivirus infection, Overexpression plasmids of PKM2 and Twist1 were obtained from TsingkeBio (Beijing, China). Briefly, packaging plasmids were co‐transfected with viral vectors into HEK‐293T cells utilizing Lipofectamine 3000 (Thermo Fisher Scientific) on the basis of manufacturer's instructions. 48 h after transfection, the medium of HEK‐293T cells were collected and centrifuged for 15 min at 3000 rpm. Supplemented with 15 µg mL^−1^ polybrene (SolarBio), purified medium were added to endothelial cells. Eventually, the infected cells were selected employing 2 µg mL^−1^ puromycin for 24 h.

### Color of Culture Medium Observation

Phenol red was added to the endothelial cell medium as an indicator that provided a visual assessment of the pH variation of the medium. Phenol red appears red at a pH of 7.4 and turns red to yellow as the pH decreases, indicating the continuous accumulation of acidic metabolites. In brief, endothelial cells were seeded at the same confluence, and the fresh medium was replaced before the treatment of endothelial cells. Several days after treatment, once the color of the hypoxic group turned yellow, the colors of the culture medium were imaged simultaneously using a microscope.

### Western Blotting

In brief, tissue samples (6 mm × 6 mm) or cellular proteins were isolated from ischemic flaps or endothelial cells and were lysed with RIPA added with 100 mM PMSF on ice for 30 min. The concentration of protein was quantified using an enhanced BCA protein assay kit (Thermo Fisher Scientific). Equivalent contents of proteins were further separated by SDS‐PAGE gel electrophoresis and then transferred onto activated PVDF membranes (Millipore). PVDF membranes were blocked with 5% nonfat milk for 2 h at room temperature and were subjected to incubation of the primary antibodies at 4 °C overnight. The day after, the membranes were washed three times for 10 min utilizing TBST and then incubated with the accordant secondary antibodies for another 2 h at room temperature and washed with TBST again. The signals of protein bands were visualized employing super sensitive ECL (Meilunbio, Dalian, China) and quantified by Bio‐Rad chemiluminescence system (USA). All antibodies employed in this study were exhibited in Table S2 (Supporting Information).

### Quantitative Real‐Time PCR Analysis

Total RNA was extracted from flap tissue, HUVECs and HDMECs utilizing TRIzol reagent (Invitrogen) basing on the manufacturer's protocols. For mRNA quantification, Advance‐Fast 1st Strand cDNA Synthesis Kit (Yeasen, Shanghai, China) for cDNA synthesis and Hieff qPCR SYBR Green Master Mix (Yeasen) for further reactions were employed according to the manufacturer's instructions. The expression levels of mRNAs were normalized to those of β‐actin. The primers used were listed in Table S3 (Supporting Information).

### Masson Trichrome Staining and Hematoxylin and Eosin (H&E) Staining

Mouse flap tissues or human skin tissues were fixed utilizing 4% paraformaldehyde. Following dehydration, the tissue samples were embedded in paraffin and then were cut into 5 µm sections. The skin sections were stained using Masson trichrome stain kit (Solarbio) to measure the fibrotic degree of skin flap. For H&E staining, the skin sections were stained with hematoxylin and eosin (Solarbio) following the manufacturer's protocols. Afterwards, the skin tissue sections were observed employing a light microscope.

### Immunohistochemistry

Skin specimens from human were processed described previously. The skin sections were subjected to antigen retrieval as well as peroxidase removal. Afterwards, the sections were blocked with 10% goat serum and were subjected to incubation at 4 °C overnight using antibodies against PKM2 and Twist1. The following day, the slides were incubated with secondary antibodies and stained using a DAB staining kit (servicebio), and then were subjected to counterstaining utilizing hematoxylin.

### Co‐Immunoprecipitation Assay

In brief, equivalent amount (200 µg) of protein of skin tissue or endothelial cells were subjected to incubation of 5 µg of anti‐p300, anti–pan‐Kac, anti–pan‐Kla or anti‐Twist1 antibody at 4 °C overnight followed by added with 50 µL protein A/G agarose beads (Yeasen) and incubating for another 4 h. Afterwards, the precipitates were washed with PBS three times and boiled at 95 °C in 1× SDS loading buffer for 5 min. Then, the indicated protein were examined using specific antibodies by western blotting.

### Immunofluorescence

For immunofluorescence staining of endothelial cells, cells were fixed using 4% paraformaldehyde for 15 min and then were permeated with 0.1% tritonX‐100 for another 15 min. As for IF of skin tissues, skin specimens from mouse were processed described previously. Subsequently, the processed cell or skin sections were blocked using 10% goat serum for 1 h at room temperature and then incubated at 4 °C overnight using primary antibodies. After washed with PBS, the cells and sections were subjected to incubation of secondary antibodies (Dylight 488 or Dylight 594, Abcam) for 1 h. DAPI was employed for nuclear staining. The EDU assay was performed on endothelial cells using an EDU Cell Proliferation Kit (Beyotime) according to the manufacturer's protocols. To measure the damage degree of skin collagen, F‐CHP (Helix3) was employed under the guidance of manufacturer's instruments. All antibodies employed in this study were exhibited in Table S2 (Supporting Information).

### Mass Spectrometry

HUVECs were cultured in 10 cm dishes under hypoxia following lactate treatment. Next day, cell lysates were harvested and incubated with Twist1 antibody at 4 °C overnight to pull down Twist1 binding proteins. Lactylated proteins were pulled down using Twist1 antibody. Electrophoresis was carried out and Coomassie Blue staining solution was employed to visualize proteins on the gel. The gel with a molecular weight of 15–30 kD were subjected to mass spectrometry detection. The peptide samples were isolated and detected using Nano source followed by Q Exactive HF‐X mass spectrometer. The electrospray voltage set was 2.0 kV, and intact peptide samples were tested in the Orbitrap at a resolution of ≈60 000. The peptide samples were then taken for MS/MS utilizing NCE applied as 27 and the obtained fragments were then tested in the Orbitrap at a resolution of ≈30 000. The Mass Spectra were subsequently acquired in data‐dependent scan mode included selection of the 20 most abundant precursor ions of each MS spectrum for MS/MS analysis with 20s dynamic exclusion.

### Luciferase Assay

To clarify the influence of Twist1 on activity of *TGFB1* responsive element under hypoxia following lactate treatment, the binding sequences, together with their mutations, were synthesized and further inserted into pGL3 luciferase reporter vector. The luciferase activities were measured following normalizing with Renilla luciferase activity respectively. The specific binding sequence were shown in Table S4 (Supporting Information).

### Generation of Twist1‐KO HUVEC

For Twist1 KO, two single guide RNAs (sgRNAs) targeting the first exon of Twist1 were selected according to the published sgRNA library. The specific sgRNA sequences were listed as follow: sgTWIST1‐1: 5′‐GGCCGGCGAGACTGGCGAGC‐3′; sgTWIST1‐2: 5′‐CTGTCGTCGGCCGGCGAGAC‐3′. Synthesized oligos for two targeting sequences were annealed and ligated to the vector. The plasmids including respective sgRNA sequence were then transfected into endothelial cells using Lipofectamine 3000. The day after transfection, puromycin were employed to filtrate the transfected cells. After selection, cells were diluted and then incubated in 96‐well culture plate. Single clone selection was carried out 2 weeks later and selected Twist1 KO clones were verified by PCR and Sanger sequencing.

### Measurements of Extracellular Acidification Rate (ECAR), Proton Efflux Rate (PER) and Oxygen Consumption Rate

Glycolysis Stress, Glycolytic Rate and Mito Stress assays were conducted utilizing the XF96 Extracellular Flux Analyzer (Agilent, CA, USA). In brief, processed endothelial cells were harvested and seeded in 96‐well Seahorse XF cell culture microplates at 1.5 × 10^4^ cells per well and were placed in an incubator with 5% CO_2_ at 37 °C overnight. For oxygen consumption rate (OCR) measurements, 1 hour before the test, the culture medium was replaced with mitochondrial stress assay buffer by supplementing Seahorse XF Base Medium, which contained 10 mM glucose, 2 mM glutamine and 1 mM pyruvate. Then the culture microplate was incubated in a CO2‐free incubator at 37 °C. A series of modulators of respiration were injected into the microplate in turn by an XF sensor cartridge to achieve ultimate concentrations of oligomycin at 1.5 µM, FCCP at 0.5 µM and rotenone/antimycin A (Rot/AA) at 0.5 µM, following the manufacturer's guideline. For ECAR measurements, the culture medium was replaced with Glycolysis Stress assay buffer medium by supplementing the Seahorse XF Base Medium, which contained 2 mM glutamine 1 h before the test. And the culture microplate was similarly incubated in a measuring incubator without CO2 input at 37 °C. A series of selective inhibitors and substrates were further injected into the microplate in turn by an XF sensor cartridge to achieve ultimate concentrations of glucose at 10 mM, oligomycin at 1.0 µM and 2‐deoxy‐glucose (2‐DG) at 50 mM, following the manufacturer's guideline. For proton efflux rate measurement, 1 h before the test, the culture medium was replaced with Seahorse XF Glycolytic Rate Assay Medium containing 10 mM glucose, 2 mM glutamine and 1 mM pyruvate 1 h before the test. Then the samples were incubated in a CO2‐free incubator at 37 °C. Next, Compounds were injected into the microplate in turn by an XF sensor cartridge to achieve final concentrations of Rot/AA at 0.5 µM and 2‐DG at 50 mM according to the manufacturer's protocol. The results of ECAR and OCR were analyzed using Seahorse XFe 96 Wave software.

### Analysis of Glucose Uptake and Lactate Production

To determine glucose uptake in endothelial cells, Glucose Uptake Colorimetric Assay Kit (Biovision) was employed following the manufacturer's instruments. Endothelial cells were seeded in 96‐well plates at 1200 cells/well. The cells were pre‐incubated with 100 µl KRPH buffer containing 2% BSA for 50 min to be starved for glucose. To activate the glucose transporter, 1 µM insulin was employed to stimulate endothelial cells, which were further subjected to 2‐DG and were incubated for 20 min. After a series of reactions, absorbance of samples was measured and calculated at 412 nm using a microplate reader. Lactate Assay Kit II (Sigma‐Aldrich) was employed to measure the serum, flap tissue and endothelial cell lactate levels following the manufacturer's protocols. Mice serum was extracted from the supernatant liquid of congealed whole blood after centrifuging at 3000 rpm at 4 °C for 15 min. For serum measurement, a 1.5 µL mouse serum sample was added with 48.5 µL Lactate Assay buffer in 96‐well plates, and then the sample was mixed with equivalent volume (50 µL) of Reaction mixes by pipetting and was subjected to incubation for 30 min at room temperature, and the absorbance of sample was detected at 450 nm. To prepare flap tissue or endothelial cell samples, samples were subjected to homogenization in 4 volumes using the Lactate Assay Buffe and were centrifuged. Then the soluble fraction was accessible to assay.

### Collagen Gel Contraction Assay

To estimate the fibroblast‐like phenotype of endothelial cells, collagen gel contraction assay was conducted. Treated endothelial cells were seeded into collagen type I (Yeasen, Shanghai, China), and the mixtures were added to 48‐well plates with density of 1×10^5^ cells/well. Following incubation for 15 min with 5% CO_2_ with 5% CO_2_ until gelled, serum‐free DMEM was subsequently added on the top of the gel. After collagen gel detachment, the area of gel was calculated utilizing ImageJ.

### Cell Migration Assay

To determine the migration capability of endothelial cells, transwell migration assay was applied. Briefly, processed endothelial cells were resuspended in serum‐free medium and seeded into the upper chambers of 24‐well culture plates. The lower chambers were filled with complete medium. After 24 h incubation, the lower side of the chambers were washed gently using PBS and further fixed with 4% paraformaldehyde for 20 min. Whereafter, cells were stained using 0.1% crystal violet reagent (Beyotime) for 15 min and were photographed by microscopy.

### Tube Formation Assay

The angiogenesis capability of endothelial cells was determined utilizing Matrigel (Corning). After Matrigel gelled in a 96‐well culture plate, endothelial cells were treated with Calcein AM (Beyotime) and seeded into the top of the Matrigel with 2 × 10^4^ cells per well. Following incubation for 6 h, capillary‐like structure was captured, and the number of formed tubes was calculated using ImageJ.

### Chromatin Immunoprecipitation (ChIP)‐qPCR

To assess whether Twist1 bind to the promoter of TGF‐β, ChIP assays were performed using a ChIP kit (ab500, abcam) following manufacturer's protocols. For ChIP assay, endothelial cells were fixed using paraformaldehyde (1% final concentraction) for 15 min to crosslink protein to DNA. Nuclear extracts were further isolated and subjected to sonication to promote chromatin to fragments. The chromatin fragmented DNA was immunoprecipitated with anti‐Twist antibody (ab50887, abcam) or anti‐igG antibody and then subjected to qPCR analysis. The sequence of primer of TGF‐β for Twist1 is: Foward 5′‐CTCTCCCGCAGACGGAATAC‐3′; Reverse 5′‐CGGGAAGTTAGCTCACCGTT‐3′.

### Statistical Analyses

All the data in this study were presented as mean ± standard deviation (SD). Data were analyzed utilizing two‐tailed unpaired Student's t‐test, one‐way analysis of variance (ANOVA) or two‐way ANOVA followed by Tukey's procedure, and *P* < 0.05 was deemed statistically significant between groups.

## Conflict of Interest

The authors declare no conflict of interest.

## Author Contributions

Y.X., X.M. and W.N. contributed equally to this work. W.G, Y.W, L.W. performed conceptualization; Y.X, X.M, Y.W. performed methodology; Y.X, L.W, W.N. performed investigation; Y.X, Z.L, L.Z. performed visualization; W.G, Y.W, L.W. performed funding acquisition; W.G, Y.W, L.W, Y.L, and N.Y. performed project administration; Z.D, T.Y, Z.C. performed supervision; Y.X, X.M, Y.W. wrote the original draft; Y.X, X.M, Y.W, L.S, H.W. wrote, reviewed and edited.

## Supporting information



Supporting Information

## Data Availability

The data that support the findings of this study are available from the corresponding author upon reasonable request.
