# Supplementary material for: PKM2‐Driven Lactate Overproduction Triggers Endothelial‐To‐Mesenchymal Transition in Ischemic Flap via Mediating TWIST1 Lactylation
Source: Adv Sci (Weinh). 2024 Oct 30;11(47):2406184. doi: 10.1002/advs.202406184 (PMC11653614; doi:10.1002/advs.202406184)
Supplement: Supplementary file 1 — Supporting Information [file ADVS-11-2406184-s001.docx]

**
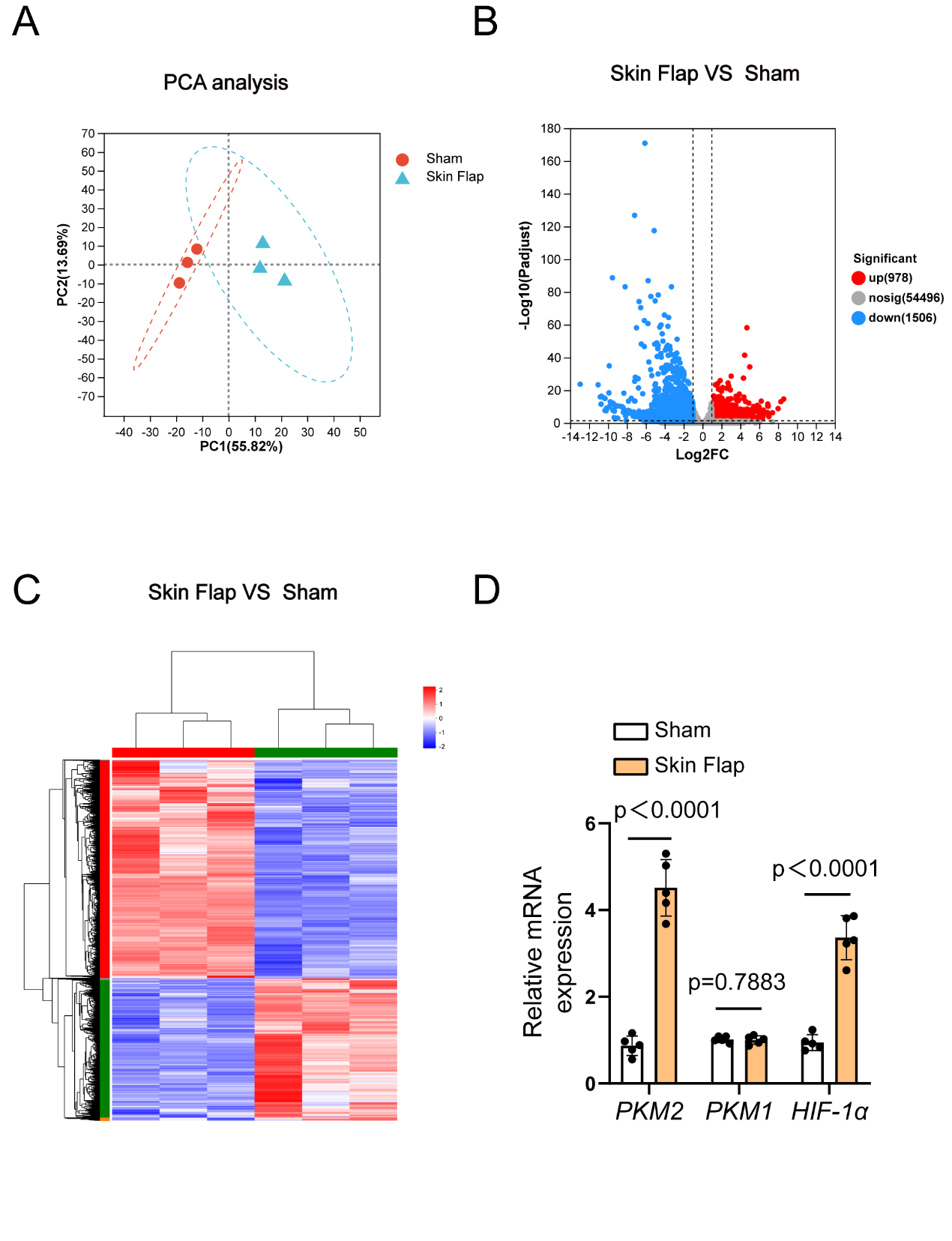
**

**Figure S1**

**Characterization of PKM2 in skin flap model.** (**A**) Quality control between the skin from sham and skin flap mice in overall RNA-seq (n=3). (**B**) differentially expressed genes in a volcano map. (**C**) differentially expressed genes in a heatmap. (**D**) mRNA levels of *PKM2*, *PKM1* and *HIF-1α* in the skin from sham and skin flap mice (n=5). Accurate *P*-values are listed in the figures. Data is presented as mean±S.D. (D), unpaired two-tailed *t* test.


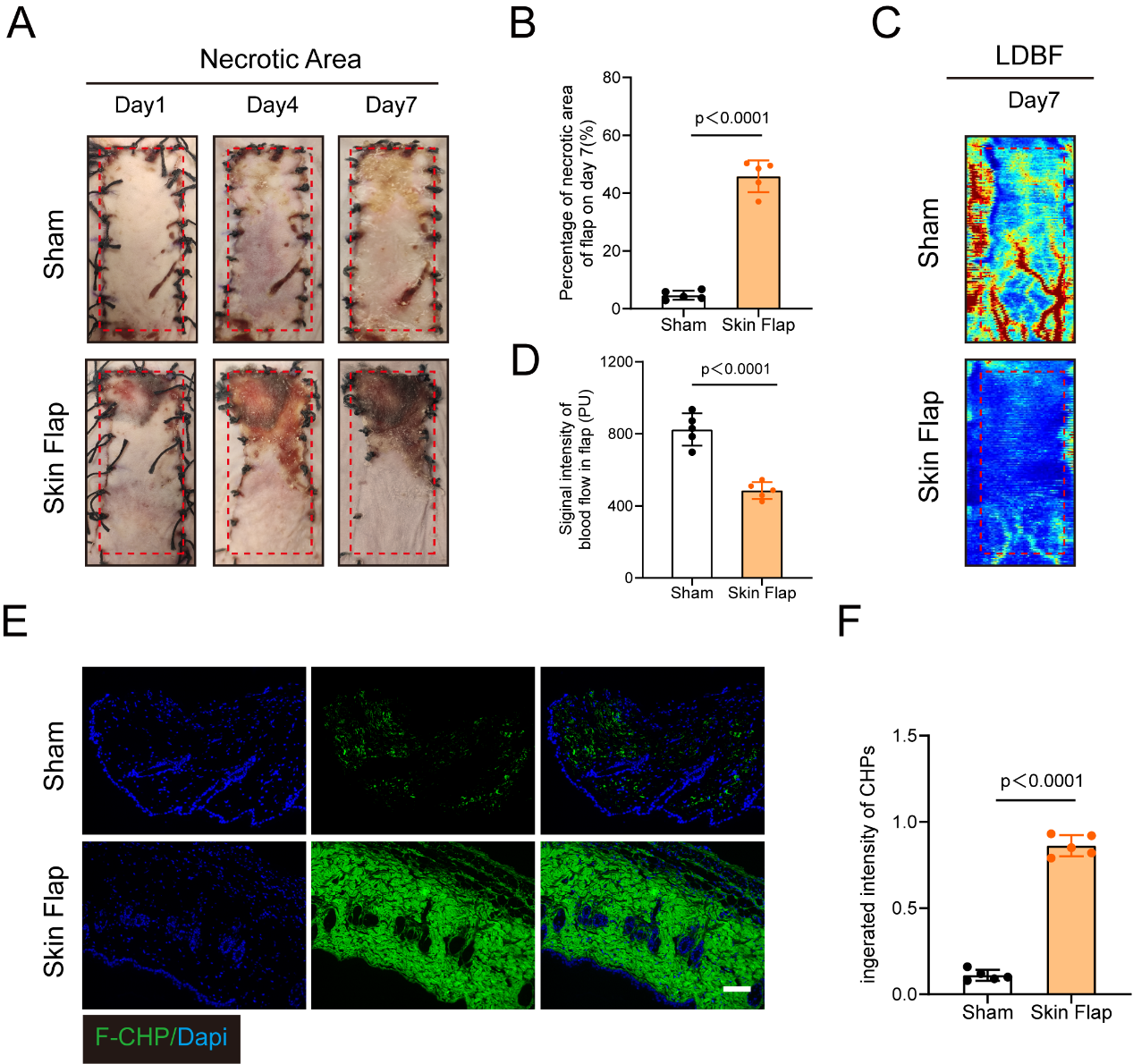


**Figure S2**

**Identification of ischemic flap-mediated necrosis.** (**A**) Photograph of random-pattern skin flaps on sham and skin flap mice at different times (1, 4 and 7 days after surgical operation). (**B**) Comparison of the survival area among the three groups on postoperative day 7 (n=5 per group). (**C**) LDBF of random-pattern skin flaps on mice on postoperative day 7 in the above group. (**D**) Comparison of signal intensity of blood flow in random-pattern skin flaps among the three groups on postoperative day 7 (n=5). (E) F-CHP staining for the evaluation of damaged collagen in the skin from sham and skin flap mice on postoperative day 7 (n=5). Scale bar: 150 μm. (**F**) Comparison of the integrated intensity of F-CHP between the two groups (n=5 per group). Accurate *P*-values are listed in the figures. Data is presented as mean±S.D. (B, D and F), unpaired two-tailed *t* test.


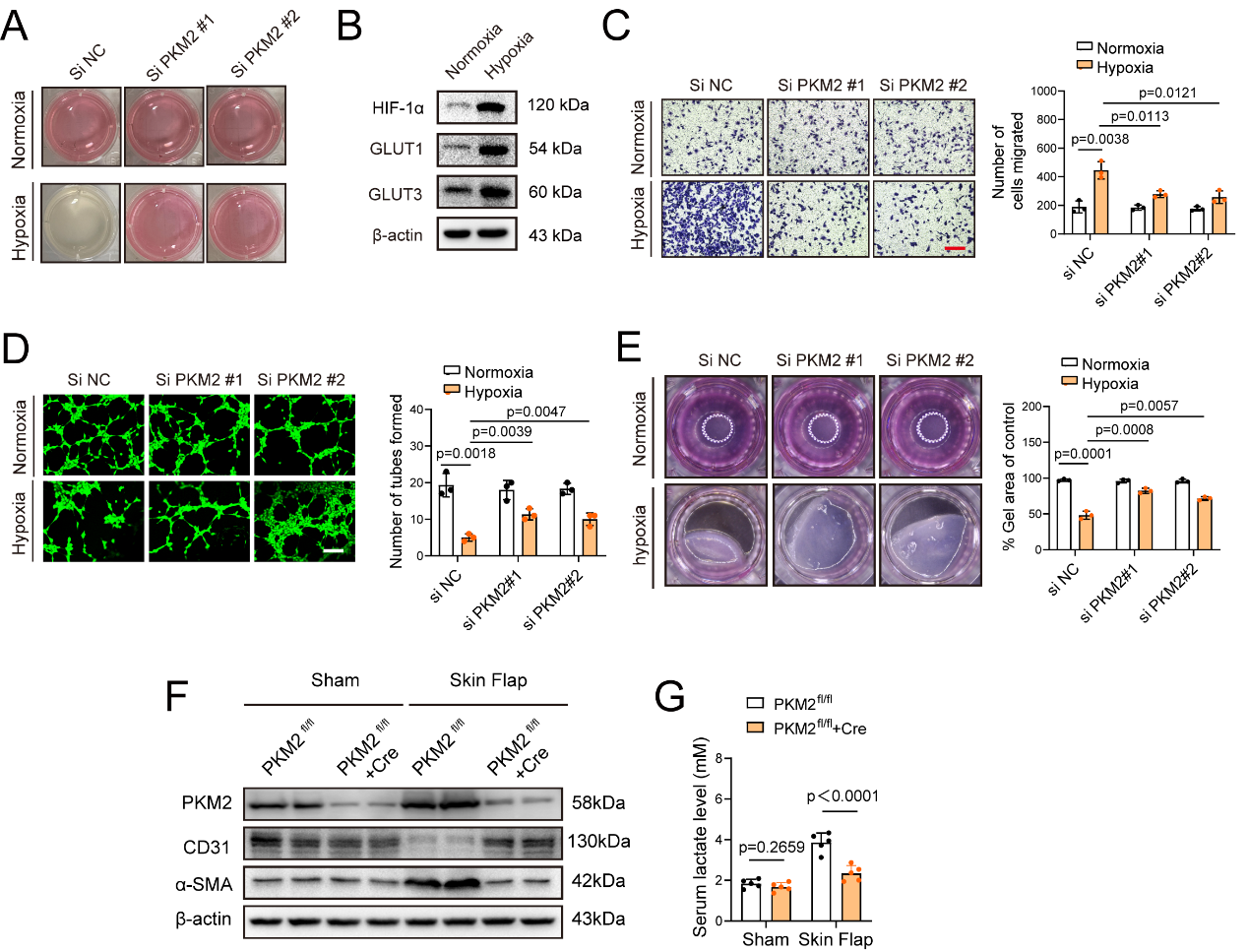


**Figure S3**

**PKM2 depletion reverses fibrotic response on endothelial cells following hypoxia/flap ischemia.** (**A**) PKM2 knockdown after hypoxic challenge stimulated acidic metabo lite accumulation as evidenced by the color of the medium. (**B**) Western blotting detection of HIF-1α, GLUT1 and GLUT3 expression under normoxia or hypoxia. (**C**) Migration capability of endothelial cell was measured by transwell assay (n=3). Scale bar: 200 μm. (**D**) Angiogenesis of endothelial cell was detected by tube formation assay (n=3). Scale bar: 200 μm. (**E**) Endothelial cell contractility was determined by collagen gel contraction assay (n=3). (**F**) Western blotting detection expression levels of PKM2, endothelial marker CD31 and mesenchymal marker α-SMA in the skin from PKM2*^fl/fl^* or PKM2*^fl/fl^*, Tie2-Cre mice that underwent skin flap surgery or sham. (**G**) Measurement of serum lactate levels from the above groups on postoperative day 7 (n=5). Accurate *P*-values are listed in the figures. Data is presented as mean±S.D. (C-E), Two-way ANOVA; (G), unpaired two-tailed *t* test.


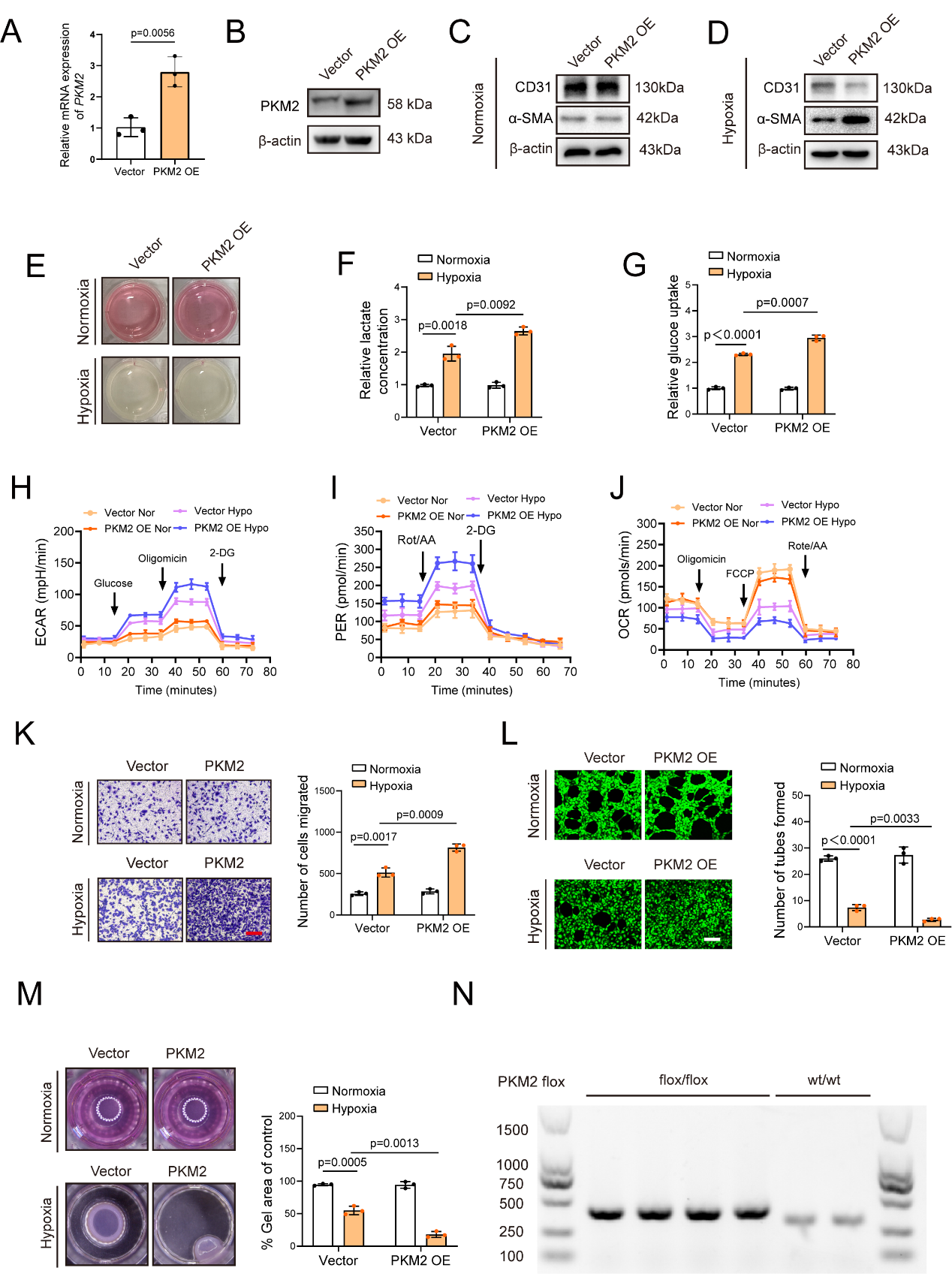


**Figure S4**

**Direct regulation impacts of PKM2 on endothelial cells.** **HUVECs were transfected with PKM2 overexpression plasmid after hypoxic or normoxic challenge.** (**A**-**B**) Overexpression of PKM2 in HUVECs using qRT-PCR and western blotting. (**C-D**) Western blotting detection of endothelial marker CD31 and mesenchymal marker α-SMA under normoxia or hypoxia. (**E**) PKM2 overexpression after hypoxia challenge stimulated acidic metabolite as evidenced by the color of the medium. (**F-G**) PKM2 overexpression following hypoxia decreased glucose uptake and lactate production in HUVECs (n=3). (**H-J**) ECAR, glycolytic rate and OCR assays of HUVECs cultured and transfected as in panel. (**K**) Migration capability of endothelial cell was measured by transwell assay (n=3). Scale bar: 200 μm. (**L**) Angiogenesis of endothelial cell was detected by tube formation assay (n=3). Scale bar: 200 μm. (**M**) Endothelial cell contractility was determined by collagen gel contraction assay (n=3). (**N**) Genotyping of *PKM2^fl/fl^* and PKM2^wt/wt^ determined by PCR. Accurate *P*-values are listed in the figures. Data is presented as mean±S.D. (A), unpaired two-tailed *t* test; (F-G and K-M), Two-way ANOVA.


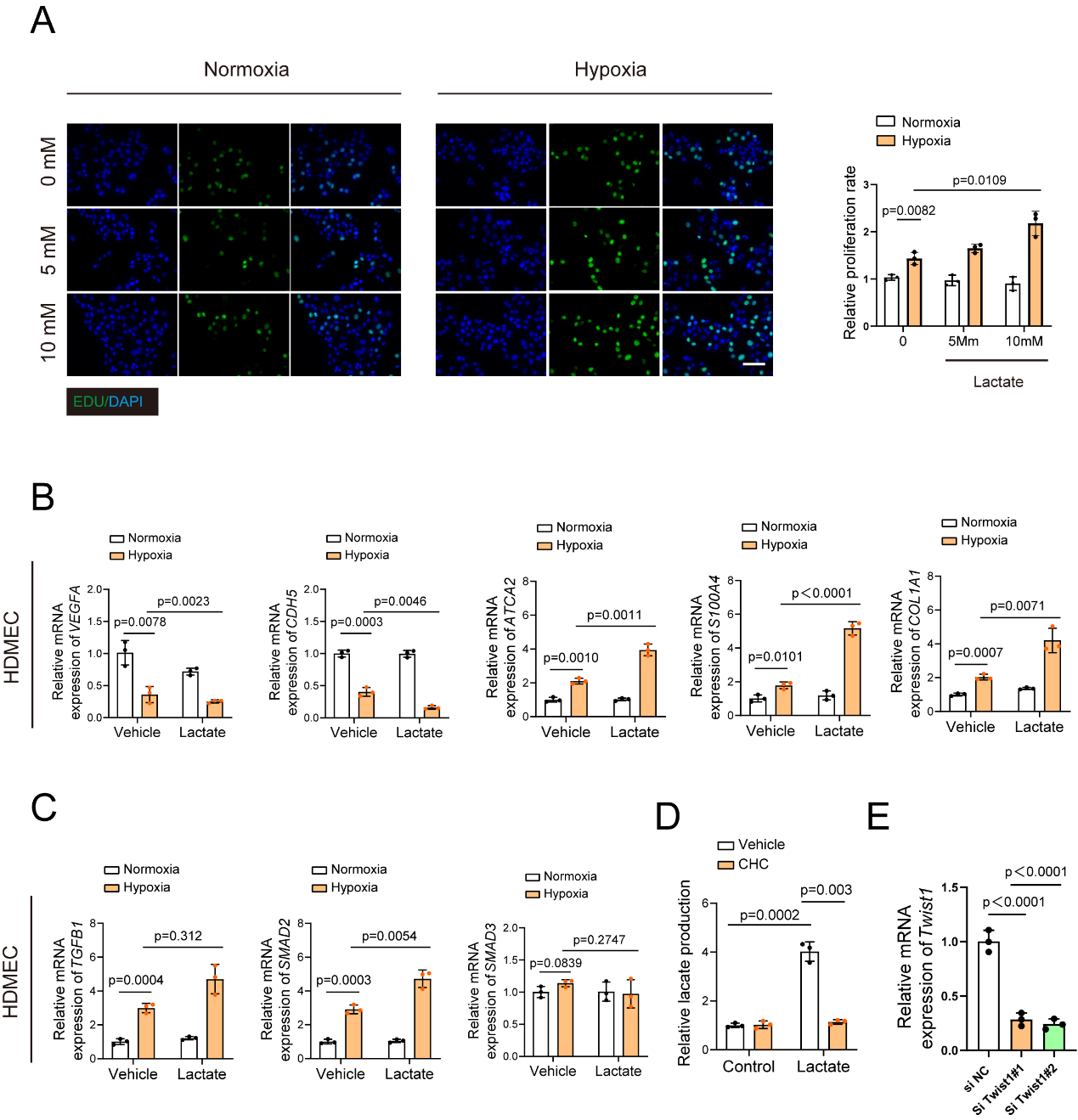


**Figure S5**

**Lactate regulates endothelial cell proliferation after hypoxia.** (**A**) HUVECs were administrated with lactate (5 or 10 mM) followed by normoxic or hypoxic challenge. Proliferation ability of endothelial cells was measured using EDU assay (n=3). Scale bar: 50 μm. (**B**) HDMECs were administrated with lactate (5 or 10 mM) followed by normoxic or hypoxic challenge. The mRNA levels of *VEGFA*, *CDH5*, *ATCA2*, *S100A4* and *COL1A1* were detected using qRT-PCR (n=3). (**C**) HDMECs were treated as described above. qRT-PCR was employed to measure the mRNA expression of *TGFB1*, *SMAD2* and SMAD3 (n=3). (**D**) HUVECs were treated with the lactate transporter MCT inhibitor α-cyano-4hydroxycinnamate (CHC) before lactate treatment. Relative intracellular lactate levels were detected (n=3). (E) Knockdown efficiency of Twist1 was evaluated by qRT-PCR in HUVECs (n=3). Accurate *P*-values are listed in the figures. Data is presented as mean±S.D. (A-D), Two-way ANOVA; (E), One-way ANOVA.

**
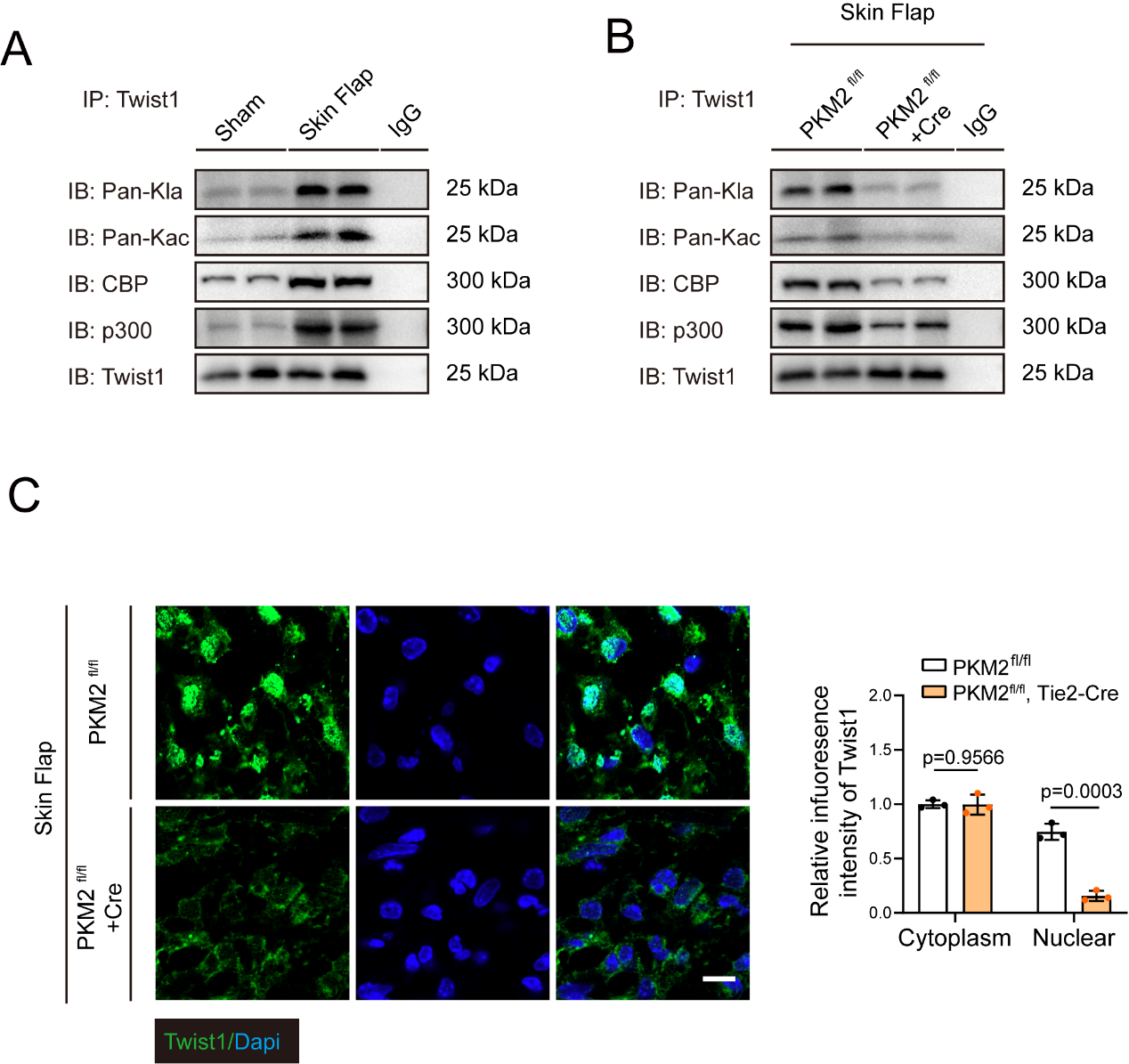
**

**Figure S6**

**Endothelial specific PKM2 deletion attenuated Twist1 nuclear translocation and lactylation.** (**A**) Lactylation and acetylation of Twist1, and the interaction between Twist1 and p300/CBP in the skin from sham and skin flap mice on postoperative day 7 were examined using Immunoprecipitation (IP). (**B**) Lactylation and acetylation of Twist1, and the interaction between Twist1 and p300/CBP in the skin from *PKM2^fl/fl^* and *PKM2^fl/fl^*, Tie2Cre mice on postoperative day 7 were examined using Immunoprecipitation (IP). (**C**) Immunofluorescence staining of Twist1 of the dermal layer in the skin from *PKM2^fl/fl^* and *PKM2^fl/fl^*, Tie2Cre mice on postoperative day 7 (n=5). Scale bar: 10 μm. Accurate *P*-values are listed in the figures. Data is presented as mean±S.D. (C), unpaired two-tailed *t* test.


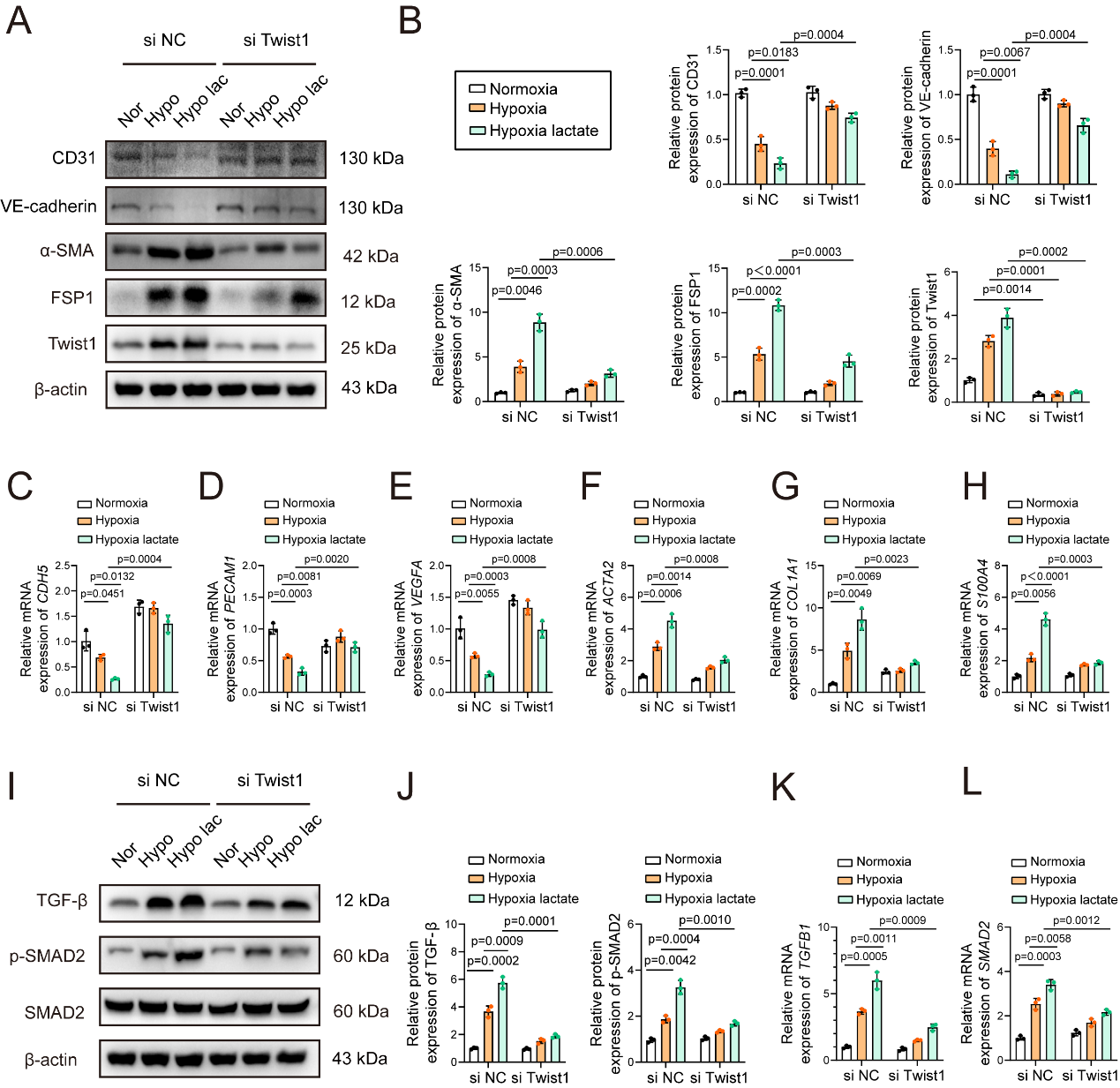


**Figure S7**

**Suppression of Twist1 ameliorated endothelial cell EndoMT an attenuated TGF-β/Smad2 activation.** HUVECs were transfected with siRNA specific for Twist1 (siTwist1). Negative control siRNA served as control (siNC). Twenty-four hours after transfection, cells were stimulated with lactate (10 mM) followed by hypoxic challenge. (**A-B**) Western blotting measurement of endothelial marker VE-cadherin, CD31 and mesenchymal marker FSP1 and α-SMA (n=3). (**C-H**) qRT-PCR was employed to measure the mRNA expression of *PECAM1*, *VEGFA*, *CDH5*, *S100A4, ACTA2* and *COL1A1*(n=3). (**I-J**) Western blotting measurement of TGF-β and p-SMAD2 (n=3). (**K-L**) qRT-PCR was employed to measure the mRNA expression of *TGFB1* and *SMAD2* (n=3). Accurate *P*-values are listed in the figures. Data is presented as mean±S.D. (B-H and J-L), Two-way ANOVA.

*
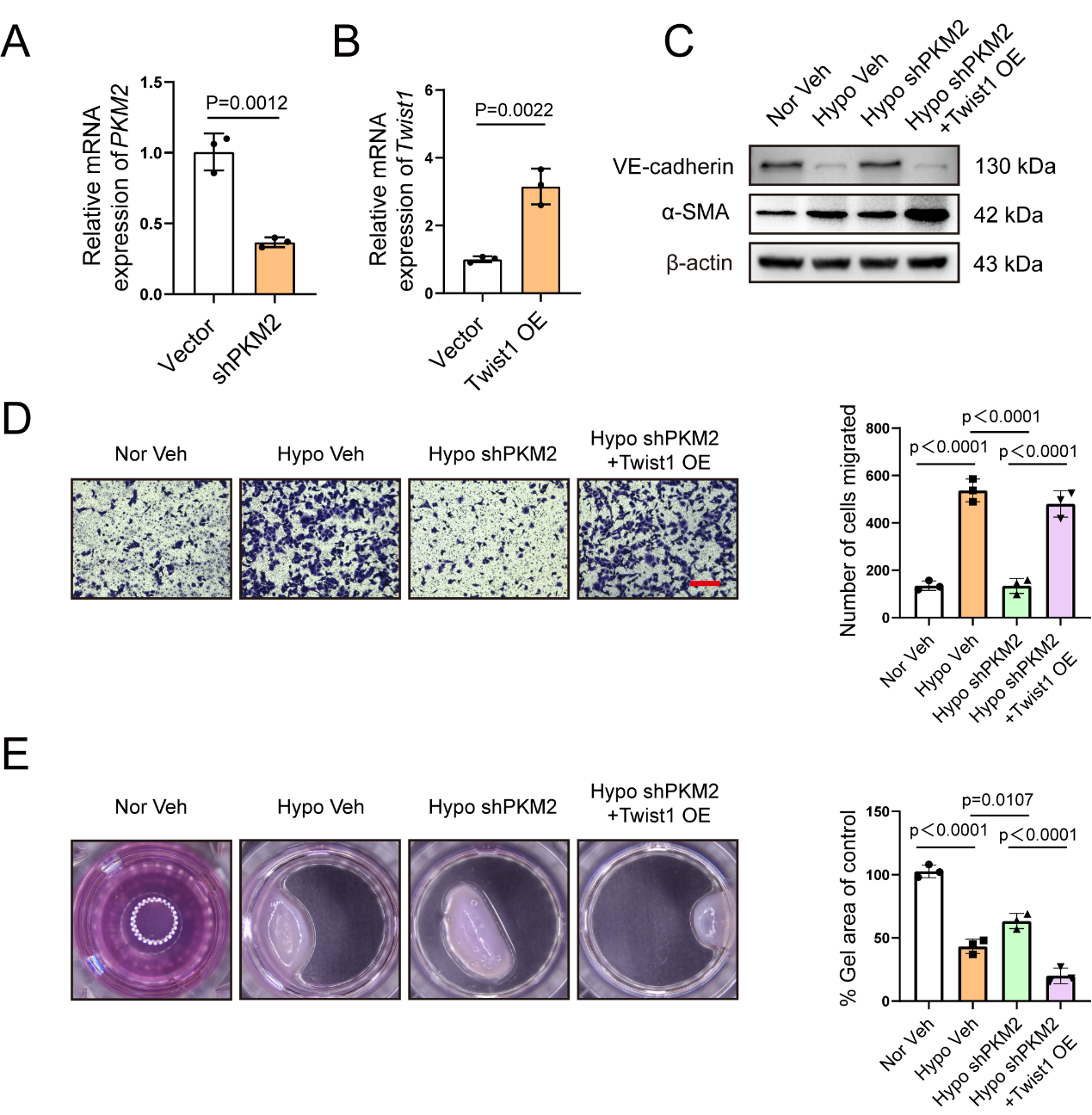
*

**Figure S8**

**Increased Twist1 promoted EndoMT and restored specific deletion of endothelial PKM2-mediated ischemic flap survival.** HUVECs were transfected with shRNA specific for PKM2, Twist1 overexpression plasmid or PKM2 shRNA and Twist1 overexpression plasmid. Negative control plasmid served as control (Vector). Twenty-four hours after transfection, indicated cells were subjected to hypoxic challenge. (**A**) qRT-PCR analysis of knockdown efficiency of shPKM2 (n=3). (**B**) qRT-PCR analysis of overexpression efficiency of Twist1 OE (n=3). (**C**) Western blotting measurement of endothelial marker VE-cadherin and mesenchymal marker α-SMA in endothelial cells. (**D**) Migration capability of endothelial cell was measured by transwell assay (n=3). Scale bar: 200 μm. (**E)** Endothelial cell contractility was determined by collagen gel contraction assay (n=3). Accurate *P*-values are listed in the figures. Data is presented as mean±S.D. (A-B and D-E), unpaired two-tailed *t* test.


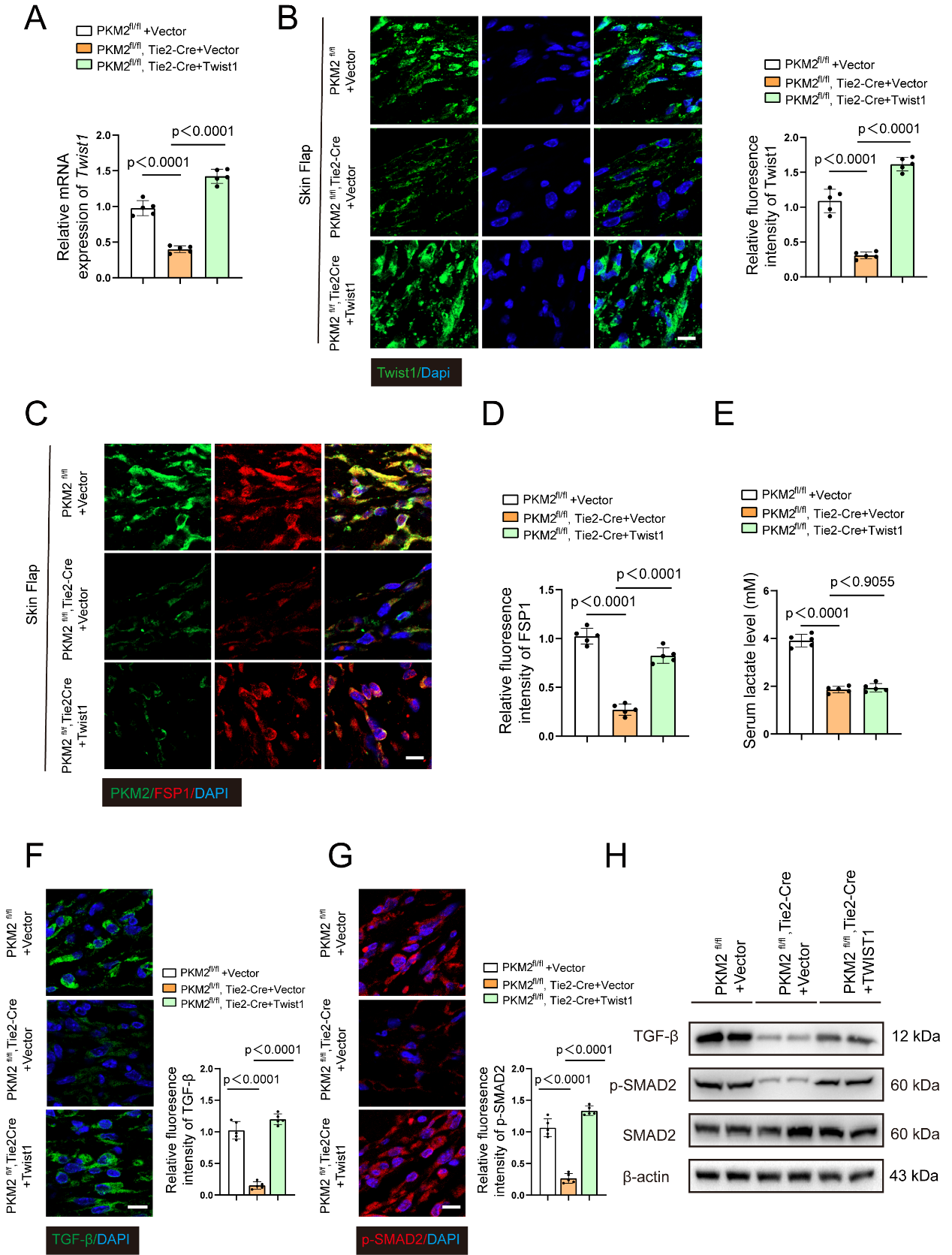


**Figure S9**

**Twist1 promoted EndoMT progression and activation of TGF-β/Smad2 signaling followed by endothelial specific PKM2 deletion in ischemic flap.** *PKM2^fl/fl^*, and *PKM2^fl/fl^*, Tie2-Cre mice were subjected to skin flap surgery and were sacrificed on postoperative day 7 (**A**) qRT-PCR measurement of overexpression efficiency of Twist1 AAV in skin from *PKM2^fl/fl^*, and *PKM2^fl/fl^*, Tie2-Cre mice that underwent skin flap surgery accompanied with Twist1 AAV or vector injection (n=5). (**B**) Immunofluorescence staining of Twist1 of the dermal layer in skin from *PKM2^fl/fl^*, and *PKM2^fl/fl^*, Tie2-Cre mice that underwent skin flap surgery accompanied with Twist1 AAV or vector injection (n=5). (**C-D**) Immunofluorescence co-staining of PKM2 and FSP1 of the dermal layer in skin from above groups (n=5). (**E**) Serum lactate detection using a commercial lactate assay kit (n=5). (**F-G**) Immunofluorescence staining of TGF-β and p-SMAD2 of the dermal layer in skin from above groups (n=5). (**H**) Western blotting measurement of TGF-β, SMAD2 and p-SMAD2 of the dermal layer in skin from above groups. Accurate *P*-values are listed in the figures. Data is presented as mean±S.D. (A-B and D-G), unpaired two-tailed *t* test.

Table S1 Sequences of siRNAs

| Human PKM2 siRNA#1 | AGGCAGAGGCUGCCAUCUATT |
| --- | --- |
| Human PKM2 siRNA#2 | CCAUAAUCGUCCUCACCAATT |
| Human Twist1 siRNA#1 | GGUGUCUAAAUGCAUUCAUTT |
| Human Twist1 siRNA#2 | AUGAAUGCAUUUAGACACCTT |

Table S2 Antibodies employed in this study

| anti-CD31 antibody | Abcam (ab93283) |
| --- | --- |
| anti-VE-cadherin antibody | Affinity (AF6265) |
| annti-FSP1 antibody | Abcam (ab197896) |
| anti-α-SMA antibody | Proteintech (67735-1-lg) |
| anti-Collagen1a1 antibody | Cell Signaling Technology (91144S) |
| anti–TGF-β antibody | Cell Signaling Technology (3711S) |
| anti–β-actin antibody | Fudebio (FD0060) |
| anti–Lamin B1 antibody | Abcam (ab16048) |
| anti-PKM1 antibody | Proteintech (15821-1-AP) |
| anti-PKM2 antibody | Proteintech (60268-1-lg) |
| anti- HIF-1α antibody | Abcam (ab308433) |
| anti–p-SMAD2 antibody | Cell Signaling Technology (18338T) |
| anti–p-SMAD3 antibody | Huabio (ET1609-4) |
| anti-SMAD2/3 antibody | Cell Signaling Technology (8685T) |
| anti-SMAD2 antibody | Cell Signaling Technology (5339T) |
| anti- -L-Lactyl Lysine antibody | PTM BIO (PTM-1401) |
| anti-Acetyl-Lysine | Abclonal (A2391) |
| anti-CBP antibody | Cell Signaling Technology (7389S) |
| anti-p300 antibody | Cell Signaling Technology (86377S) |
| anti-twist1 (pSer68) antibody | Abcam (ab187008) |
| Anti- Phospho-PKM2 (Tyr105) antibody | Cell Signaling Technology (3827S) |
| Anti-TWIST1 antibody | Proteintech (25465-1-AP) |

Table S3 Primer sequences employed in this study

| ACTB  (human) | F: AGAGCTACGAGCTGCCTGAC  R: AGCACTGTGTTGGCGTACAG |
| --- | --- |
| PKM2  (human) | F: GCTGCCATCTACCACTTGC  R: CCAGACTTGGTGAGGACGATT |
| CDH5  (human) | F: CGCAATAGACAAGGACATAAC  R: TATCGTGATTATCCGTGAGG |
| PECAM1  (human) | F: AGATACTCTAGAACGGAAGG  R: CAGAGGTCTTGAAATACAGG |
| VEGFA  (human) | F: AGGGCAGAATCATCACGAAGT  R: AGGGTCTCGATTGGATGGCA |
| ACAT2  (human) | F: AGATCAAGATCATTGCCCC  R: TTCATCGTATTCCTGTTTGC |
| S100A4  (human) | F: AAGTTCAAGCTCAACAAGTC  R: CAGCTTCATCTGTCCTTTTC |
| COL1A1  (human) | F: GCTATGATGAGAAATCAACCG  R: TCATCTCCATTCTTTCCAGG |
| TGFB1  (human) | F: AACCCACAACGAAATCTATG  R: CTTTTAACTTGAGCCTCAGC |
| SMAD2  (human) | F: CAGTTTTGCCTCCAGTATTAG  R: AGTGAGTATAGTCATCCAGAG |
| SMAD3  (human) | F: TGGACGCAGGTTCTCCAAAC  R: CCGGCTCGCAGTAGGTAAC |
| Twist1  (human) | F: GTCCGCAGTCTTACGAGGAG  R: GCTTGAGGGTCTGAATCTTGCT |
| ACTB  (mouse) | F: CCACCATGTACCCAGGCATT  R: CCGATCCACACAGAGTACTT |
| PKM2  (mouse) | F: CGCCTGGACATTGACTCTG  R: GAAATTCAGCCGAGCCACATT |
| PKM1  (mouse) | F: CGAGCCTCAAGTCACTCCAC  R: GTGAGCAGACCTGCCAGACT |
| Twist1  (mouse) | F: GGACAAGCTGAGCAAGATTCA  R: CGGAGAAGGCGTAGCTGAG |
| Hif1a  (mouse) | F: GATGACGGCGACATGGTTTAC  R: CTCACTGGGCCATTTCTGTGT |

Table S4 The binding sequences for Luciferase activity assays are as follows:

| Resource Sequence (5’-3’) | |
| --- | --- |
| *TGFB1*-ChIP1 | GCAAGCCCAAGGTGGAGCAGCTGTCCAACATGATCGTGCGCTCCT |
| *TGFB1*-ChIP2 | CACGGAGAAGAACTGCTGCGTGCGGCAGCTGTACATTGACTTCCG |
| *TGFB1*-ChIP1 mutation | GCAAGCCCAAGGTGGAGTGATCATCCAACATGATCGTGCGCTCCT |
| *TGFB1*-ChIP2 mutation | CACGGAGAAGAACTGCTGCGTGCGGTGATCATACATTGACTTCCG |
| *TGFB1*-ChIP1-ChIP2 | GCAAGCCCAAGGTGGAGCAGCTGTCCAACATGATCGTGCGCTCCT CACGGAGAAGAACTGCTGCGTGCGGCAGCTGTACATTGACTTCCG |
